# Stereoselective Halogenation of Integral Unsaturated C‐C Bonds in Chemically and Mechanically Robust Zr and Hf MOFs

**DOI:** 10.1002/chem.201505185

**Published:** 2016-02-24

**Authors:** Ross J. Marshall, Sarah L. Griffin, Claire Wilson, Ross S. Forgan

**Affiliations:** ^1^ WestCHEMSchool of ChemistryThe University of GlasgowUniversity AvenueGlasgowG12 8QQUK

**Keywords:** gas adsorption, iodine, metal–organic frameworks, postsynthetic modification, zirconium

## Abstract

Metal–organic frameworks (MOFs) containing Zr^IV^‐based secondary building units (SBUs), as in the UiO‐66 series, are receiving widespread research interest due to their enhanced chemical and mechanical stabilities. We report the synthesis and extensive characterisation, as both bulk microcrystalline and single crystal forms, of extended UiO‐66 (Zr and Hf) series MOFs containing integral unsaturated alkene, alkyne and butadiyne units, which serve as reactive sites for postsynthetic modification (PSM) by halogenation. The water stability of a Zr–stilbene MOF allows the dual insertion of both −OH and −Br groups in a single, aqueous bromohydrination step. Quantitative bromination of alkyne‐ and butadiyne‐containing MOFs is demonstrated to be stereoselective, as a consequence of the linker geometry when bound in the MOFs, while the inherent change in hybridisation and geometry of integral linker atoms is facilitated by the high mechanical stabilities of the MOFs, allowing bromination to be characterised in a single‐crystal to single‐crystal (SCSC) manner. The facile addition of bromine across the unsaturated C−C bonds in the MOFs in solution is extended to irreversible iodine sequestration in the vapour phase. A large‐pore interpenetrated Zr MOF demonstrates an I_2_ storage capacity of 279 % *w*/*w*, through a combination of chemisorption and physisorption, which is comparable to the highest reported capacities of benchmark iodine storage materials for radioactive I_2_ sequestration. We expect this facile PSM process to not only allow trapping of toxic vapours, but also modulate the mechanical properties of the MOFs.

## Introduction

Metal–organic frameworks (MOFs)^1^ are multidimensional coordination networks comprised of metal nodes separated by organic linkers that have received widespread interest over the past 10‐15 years,^2^ mainly attributable to their permanent porosity, leading to potential application in areas such as gas capture and storage,^3^ catalysis^4^ and drug delivery.^5^ The judicious choice of both the organic and inorganic constituents of MOFs enables vast opportunities for framework design,^6^ leading to materials with intrinsically variable structures^7^ and properties.^8^ Research focused on improving the stabilities of MOFs^9^ has resulted in the utilisation of high valent metal cations in the secondary building units (SBUs). Most notably, Zr^IV^ MOFs, in particular the so‐called UiO‐66 series^10^ wherein dicarboxylate ligands connect Zr_6_O_4_(OH)_4_ clusters, demonstrate increased chemical^11^ and mechanical^12^ stabilities. The synthesis of Zr MOFs^13^ has been known to be tricky; however, coordination modulation^14^ has greatly improved the ability to access both bulk microcrystalline^15^ and single crystal forms.^16^

**Figure 1:**
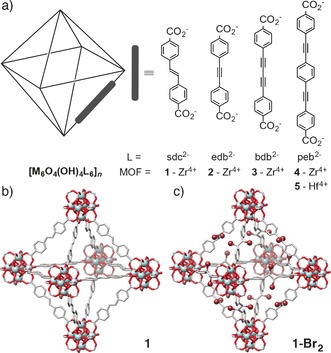
a) Abbreviation scheme of the ligands and MOFs used throughout this study. Note that **4** and **5** display two‐fold interpenetration. b) Representation of the crystal structure of **1**, which can be brominated in a SCSC manner to c) **1‐Br_2_**. Atom colouring: Zr (cyan); C (grey); O (red); Br (brown). H atoms are omitted for clarity.

The chemical stabilities of Zr MOFs make them attractive candidates for postsynthetic modification (PSM), whereby chemical transformations are performed on pre‐synthesised MOFs whilst maintaining crystallinity.^17^ PSM offers a vast array of opportunities for the functionalisation of Zr MOFs, although this has typically been limited to ligand/metal‐ion exchange,^18^ ligand metalation13a, 19 and covalent modifications of pendant functional groups.^20^ Across all MOFs, postsynthetic bromination has been attempted mainly on tethered moieties,^21^ with limited success obtained for integral units.^22^ In contrast, we recently communicated the stereoselective bromination of integral alkene and alkyne units contained within the Zr MOFs **1** and **2**, respectively, (Figure 1) in a single‐crystal to single‐crystal (SCSC) manner, resulting in a mechanical contraction of the frameworks accompanied by an increase in ligand flexibility, which in turn alters mechanical compliance.^23^

Herein we significantly broaden the scope of both the MOFs and functional groups that can be postsynthetically modified, as well as the chemical transformations that are utilised. Continuing with our interest in introducing flexibility into typically rigid UiO‐66 type MOFs,^24^ we describe the synthesis (see Supporting Information, Sections S2 and S3) of a new Zr MOF **3**, constructed from a butadiyne‐containing organic ligand, alongside a pair of analogous interpenetrated isoreticular Zr and Hf MOFs,^25^ **4** and **5**, respectively, containing an extended 4,4′‐[1,4‐phenylenebis(ethyne‐2,1‐diyl)]‐dibenzoate (peb^2−^) linker (Figure 1 a). The new MOFs are extensively characterised using a number of techniques, including single‐crystal X‐ray diffraction (SCXRD), and postsynthetic halogenation of their integral reactive sites has also been thoroughly investigated.

## Results and Discussion

Postsynthetic bromination of integral alkenes within MOFs has been highlighted as an attractive route for the stereocontrolled synthesis of bromoalkanes. Bromination of a Zn–stilbene MOF has previously been reported, however the forcing conditions required for quantitative conversion (100 °C) resulted in degradation of the MOF.22a We recently communicated the quantitative bromination of the Zr–stilbene MOF [Zr_6_O_4_(OH)_4_(sdc)_6_]_*n*_ (**1**) to [Zr_6_O_4_(OH)_4_(*meso‐*sdc‐Br_2_)_6_]_*n*_ (**1‐Br_2_**), as both bulk microcrystalline and single‐crystal material (Figure 1 b and 1c).23b Considering the harsh bromination conditions employed (neat Br_2_), we decided to investigate the use of *N*‐bromosuccinimide (NBS) as a milder, alternative brominating agent (Table 1).^26^

**Table 1:** Reaction scheme for PSM of **1** using NBS highlighting potential products, with a summary of reaction conditions investigated. Product distributions are calculated by ^1^H NMR spectroscopy of digested samples ([D_6_]DMSO/D_2_SO_4_).

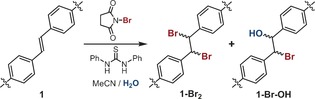								
Entry	alkene [mmol]	NBS equiv	DPT equiv	Solvent mixture [mL]		Product distribution [%]		
	in **1**			MeCN	H_2_O	**1**	**1‐Br_2_**	**1‐Br‐OH**
1	0.053	5	0	5	0	45	55	0
2	0.053	15	0	5	0	0	100	0
3	0.053	5	0.1	5	0	38	50	12
4	0.053	15	0.1	5	0	21	62	17
5	0.053	5	0	3	2	29	29	42
6	0.053	5	0.1	3	2	0	51	49
7	0.053	5	0.1	3	3	0	33	67
8	0.053	5	0.1	3	4	0	35	65
9	0.053	3	0.1	3	3	0	34	66
10	0.053	2	0.1	3	3	0	37	63
11	0.264	5	0.1	15	10	0	43	57

Addition of 15 equivalents of NBS (entry 2) was necessary to quantitatively brominate **1**. Addition of *N*,*N′*‐diphenylthiourea (DPT), a known catalyst for activation of NBS, gave small amounts of the bromohydrinated product, **1‐Br‐OH**, alongside the brominated material **1‐Br_2_** (entry 3), presumably resulting from excipient water in the reagent or solvent. Incorporation of hydroxyl units within MOFs allows pore hydrophilicity to be tuned, yet direct synthesis of such MOFs is not always possible due to coordination of the hydroxyl group to the metal ions.^27^ It is therefore surprising that few efforts have focused on the introduction of hydroxyl groups, either through protection–deprotection mechanisms^28^ or postsynthetically.^29^ To that end, reaction conditions were tailored to promote conversion to **1‐Br‐OH**, with increased amounts of NBS resulting in slightly better conversion (entry 4), but the yield of **1‐Br‐OH** remained low. Deliberate addition of water (entry 5) gave significantly more bromohydrinated product, while the combined presence of both water and DPT gave full conversion and up to 67 % bromohydrination (entries 6–8) versus bromination. Reduced quantities of NBS (entries 9 and 10) gave similar product distributions; around 60–65 % of **1‐Br‐OH** and 35–40 % of **1‐Br_2_**. Similar product distributions were obtained when the reaction was performed on a larger scale (entry 11).

Considering the similarity of bromohydrination to bromination of **1**, which results exclusively in *meso*‐sdc‐Br_2_^2−^,23b we assume that only the enantiomeric (*R*,*S*)/(*S*,*R*)‐isomers are obtained (see Supporting Information, Section S4). The fact that **1** remains crystalline during the transformation highlights its high *mechanical* stability, as it is able to withstand a change in geometry of the central carbon atoms as their hybridisation changes from sp^2^ to sp^3^. The *chemical* stability of **1** is also apparent as it retains its framework structure in the presence of significant amounts of water. The incorporation of hydroxyl groups generates a platform for subsequent functionalisation of MOFs, with alkoxide formation^30^ and incorporation of catalytic units^31^ previously described. Whilst NBS has allowed us to bromohydrinate **1**, enabling the insertion of orthogonal functionality into the MOF in one‐step, we have chosen Br_2_ as a favoured brominating agent for Zr‐MOFs bearing integral unsaturated functionalities.

**Figure 2:**
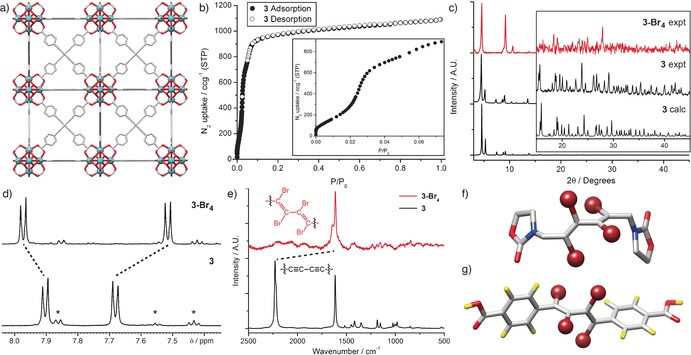
a) Representation of the single‐crystal structure of **3**. b) N_2_ adsorption isotherm at 77 K of **3**, with the insert highlighting the stepwise adsorption observed at low partial pressures. c) Comparison between predicted and experimental PXRD patterns of **3**, alongside the experimental pattern of **3‐Br_4_**. Comparisons of the d) ^1^H NMR spectra (the asterisk denotes residual benzoic acid) and e) Raman spectra of **3** and **3‐Br_4_**, with both techniques suggesting quantitative conversion. f) Crystal structure of a rare tetrabromodialkene‐containing compound (CCDC deposition RUWROR)^34^ highlighting both the vertical and horizontal displacements as a result of the steric bulk of the Br atoms. g) A molecular dynamics minimised (UFF) representation of the similarly expected arrangement of bdb‐Br_4_‐H_2_. Atom colouring: Zr (cyan); C (grey); O (red); N (blue); H (yellow); Br (brown). H atoms in parts a) and f) are omitted for clarity.

Inspired by the work of Anderson and co‐workers, in which they show that butadiyne units are highly flexible and can adopt non‐linear geometries,^32^ we decided to synthesise 4,4′‐(buta‐1,3‐diyne‐1,4‐diyl)‐dibenzoic acid (bdb‐H_2_) for incorporation into a UiO‐66 series MOF. Bulk microcrystalline and single‐crystal samples of [Zr_6_O_4_(OH)_4_(bdb)_6_]_*n*_ (**3**) were synthesised in the presence of 30 equivalents of benzoic acid (see Supporting Information, Section S3).^33^ SCXRD reveals that **3** adopts the typical UiO‐66 topology, with Zr_6_O_4_(OH)_4_ clusters connected 12‐fold by bdb^2−^ ligands (Figure 2 a) in the *Fm* $\bar{3}$ *m* space group with unit cell edge *a*=33.3694(3) Å. N_2_ adsorption isotherms performed on bulk samples at 77 K prove the permanent porosity of **3**, which displays a type I isotherm with stepwise adsorption observed at low partial pressures, characteristic of filling of the smaller tetrahedral and larger octahedral pores of UiO‐66 type MOFs (Figure 2 b). The high porosity of **3** is evident from its BET surface area of 3850 m^2^ g^−1^, in line with what was expected when compared with **2**, which contains shorter edb^2−^ connecting ligands and has a BET surface area of 3300 m^2^ g^−1^.23a

With **3** in hand, we envisaged that bromination of its butadiyne units should be possible to form **3‐Br_4_**. There exists little literature concerning the bromination of butadiynes to tetrabromodialkene units,^34^ but nonetheless the bromination of **3** was initially attempted by exposing bulk microcrystalline samples to solutions of neat Br_2_ (15 equivalents per alkyne unit) in CHCl_3_, with the transformation followed using a number of experimental techniques (see Supporting Information, Section S5).

The transformation to **3‐Br_4_** was evidenced by PXRD (Figure 2 c); excellent agreement between the predicted (from the single‐crystal structure) and experimental PXRD patterns of **3** reveal that the bulk microcrystalline samples are of excellent phase purity and, upon comparison with the brominated material **3‐Br_4_**, it is clear that a structural transition has occurred due to distinct changes in both peak positions and their relative intensities. ^1^H NMR spectra of acid‐digested samples of **3** and **3‐Br_4_** (Figure 2 d) provide evidence of quantitative bromination to a single isomer, and we assume the spectrum to comprise *trans*,*trans*‐bdb‐Br_4_‐H_2_, due to the steric restrictions imposed on the linker when bound within the MOF. The presence of only aromatic protons provides limited information and, unfortunately, ^13^C NMR spectra of digests of **3** were not possible due to the limited solubility of bdb‐H_2_. In the ^13^C NMR spectrum of **3‐Br_4_**, however, seven signals are observed, which correspond to the carbon atoms of the brominated product, alongside two small peaks around *δ*=130 ppm, which represent aromatic carbon atoms of residual benzoic acid modulator (see Supporting Information, Section S5).^33^ The number of resonances indicate only one symmetric product is formed, while the very similar values for the chemical shifts of the *trans*‐dibromoalkene carbon atoms in **3‐Br_4_** compared to the analogous MOF **2‐Br_2_** (*δ*=117.6 ppm vs. *δ*=118.2 ppm, respectively) suggests that the single species is the *trans*,*trans*‐bdb‐Br_4_‐H_2_ product; that is, the bromination of **3** occurs stereoselectively. In contrast, the liquid‐phase bromination of the dimethyl ester of the ligand (bdb‐Me_2_), using conditions similar to those employed for the bromination of **3** (neat Br_2_, CHCl_3_ solution), resulted in a ^1^H NMR spectrum containing resonances for three species, which we have assigned to be the *trans*,*trans*, *cis*,*trans* and *cis*,*cis* geometric isomers of bdb‐Br_4_‐Me_2_ (see Supporting Information, Section S6).

Raman spectroscopy (Figure 2 e) also suggests quantitative conversion, with the complete disappearance of the peak at 2230 cm^−1^, representative of the butadiyne functionality, combined with the emergence of a distinct shoulder on the alkene peak at about 1600 cm^−1^, attributable to the formation of the tetrabromodialkene moiety as **3** is brominated to **3‐Br_4_**. Excluding initial solvent loss (<200 °C), thermogravimetric analysis (TGA) in air reveals that **3** is thermally stable to about 460 °C, typical of UiO‐66 type MOFs (see Supporting Information, Section S5). The TGA profile of **3‐Br_4_** displays an additional mass loss event that we expect to correspond to debromination, but which could not be fully deconvoluted from the overall decomposition. The total mass loss between 200–550 °C (82.8 %) corresponds very closely to the theoretical mass loss (82.9 %) expected upon decomposition of **3‐Br_4_** to ZrO_2_, in contrast to the 70.7 % mass loss for **3** over the same temperature range (expected 69.3 %), which, along with bromine analysis (Br content 44.3 % calculated; 43.1 % found) and the spectroscopic data, suggests quantitative bromination.

Single crystals of **3** were suspended in CHCl_3_ and exposed to Br_2_ for four days, thereafter the CHCl_3_ was replenished several times before finally resolvating in DMF. In contrast to our solution of the crystal structures of **1‐Br_2_** and **2‐Br_2_**, it was only possible to collect unit cell parameters for **3‐Br_4_**. We would expect the lowest energy arrangement of the bdb‐Br_4_^2−^ linker to result in the tetrabromodialkene unit being geometrically frustrated both horizontally and vertically as a consequence of the steric bulk of the bromo substituents; this conformation has been observed in the crystal structure of a tetrabromodialkene analogue^34^ (Figure 2 f) and in our energy minimisation of bdb‐Br_4_‐H_2_ (Figure 2 g). As the linker sits along a linear vector in the framework, the resultant MOF **3‐Br_4_** will exhibit significant disorder. Despite this, comparison of the unit cell edges of **3** (*a*=33.3694(3) Å) and **3‐Br_4_** (*a*=32.7864(7) Å) clearly shows a mechanical contraction associated with the bdb^2−^ linker shortening upon bromination, consistent with the brominations of **1** and **2**.^23^ Unfortunately, we have found that **3‐Br_4_** is not porous and so cannot examine the mechanical modification by gas uptake. We hypothesise that the large mechanical contraction combined with the significant ligand disorder brought on by bromination may result in pore collapse.

**Figure 3:**
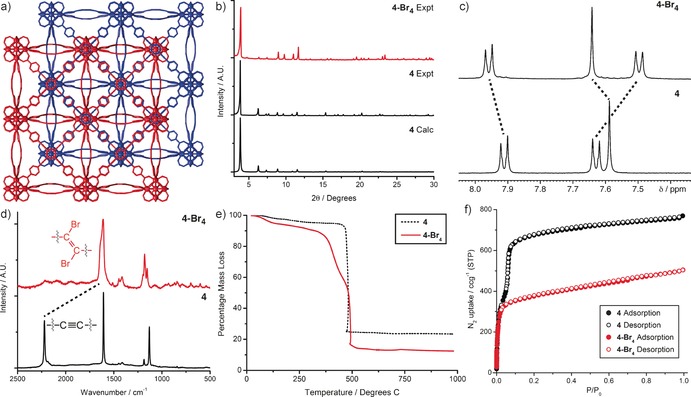
a) Packing diagram of **4** derived from its single crystal structure, with two independent frameworks coloured red and blue to highlight the interpenetrated structure. b) Comparison between predicted and experimental PXRD patterns of **4**, alongside the experimental pattern of **4‐Br_4_**. Comparisons of the c) ^1^H NMR and d) Raman spectra of **4** and **4‐Br_4_**, with both techniques suggesting quantitative conversion. e) TGA profiles highlighting the presence of a debromination step that has been introduced upon bromination of **4** to **4‐Br_4_**. f) Adsorption isotherms at 77 K highlighting the decrease in N_2_ uptake as a result of the mechanical contraction induced upon bromination of **4** to **4‐Br_4_**. Analogous data for **5** and **5‐Br_4_** can be found in the Supporting Information, Section S7.

Interpenetrated Zr MOFs containing substituted derivatives of the extended, alkyne‐containing ligand 4,4′‐[1,4‐phenylenebis(ethyne‐2,1‐diyl)]‐dibenzoate (peb^2−^), were reported in 2011.^25^ Here, we detail the synthesis of the analogous unsubstituted Zr MOF [Zr_6_O_4_(OH)_4_(peb)_6_]_*n*_ (**4**) and the Hf derivative [Hf_6_O_4_(OH)_4_(peb)_6_]_*n*_ (**5**) (See Supporting Information, Section S3).^35^ Single crystals obtained using l‐proline modulation were smaller (50 μm) than those from conventional benzoic acid modulated syntheses (100 μm), and so benzoic acid modulated crystals were analysed by SCXRD. Both **4** (Figure 3 a) and **5** crystallise in the cubic *Fd* $\bar{3}$ *m* space group with unit cell edges of *a*=39.8116(7) Å and *a*=39.806(5) Å, respectively, and are structurally similar to their functionalised analogues.^25^ The high flexibility of the peb^2−^ ligands is evident, as they bow in and out of the linear plane separating adjacent M_6_O_4_(OH)_4_ (M=Zr or Hf) clusters. Experimental PXRD patterns of bulk samples of **4** and **5** prepared by l‐proline modulation show excellent agreement (Figure 3 b) with the patterns predicted from their single‐crystal structures.

The presence of alkyne functionalities within the frameworks prompted us to attempt postsynthetic bromination of both **4** and **5**, and we have analysed the transformation using a number of techniques, which are discussed below for **4‐Br_4_** but are also described for **5‐Br_4_** in the Supporting Information, Section S7. The PXRD pattern of the brominated material, **4‐Br_4_**, reveals that crystallinity is retained during the transformation (Figure 3 b) and it is clear that peak positions have moved to slightly higher values of 2*θ* upon bromination, indicative of the conversion of alkyne to dibromoalkene units and the mechanical contraction that results.23a

The presence of a single species in the ^1^H NMR spectrum of acid‐digested **4‐Br_4_** (Figure 3 c), with altered chemical shifts relative to the parent framework, suggests that postsynthetic bromination has again occurred quantitatively and stereoselectively. Considering the geometrical constraints imposed on the ligand when bound within the MOF, we assume that the *trans*,*trans*‐peb‐Br_4_^2−^ isomer is formed exclusively. Conversely, the solution‐phase bromination of the dimethyl ester of the ligand, peb‐Me_2_ to peb‐Br_4_‐Me_2_, occurs non‐stereoselectively, with all three possible geometrical isomers (*trans*,*trans*‐peb‐Br_4_‐Me_2_, *cis*,*trans‐*peb‐Br_4_‐Me_2_ and *cis*,*cis‐*peb‐Br_4_‐Me_2_) obtained (see Supporting Information, Section S8). Raman spectra of the MOFs (Figure 3 d) suggest quantitative conversion of the alkyne to dibromoalkene units, with the complete disappearance of the alkyne peak at 2220 cm^−1^ combined with a broadening of the alkene peak at about 1600 cm^−1^ indicative of the alkene and the newly formed dibromoalkene peaks being superimposed.

Excluding solvent loss (<200 °C), TGA reveals that **4** is thermally stable to about 470 °C, typical of Zr MOFs (see Supporting Information, Section S7). The TGA profiles of **4** and **4‐Br_4_** are clearly different (Figure 3 e), with two distinct mass loss events observed for **4‐Br_4_**. Assuming that the first mass loss of **4‐Br_4_** (200–465 °C) represents debromination, there is excellent agreement between the observed mass loss of approximately 39.1 % and the theoretical Br content of 40.1 %, which, in concert with a bromine content of 41.9 % by elemental analysis, suggests quantitative bromination has been achieved. N_2_ adsorption isotherms prove the permanent porosity of **4** which has a BET surface area of 2650 m^2^ g^−1^, decreasing to 1440 m^2^ g^−1^ upon bromination to **4‐Br_4_** (Figure 3 f). The higher mass of **4‐Br_4_** cannot fully account for this decrease in gravimetric surface area, and calculated pore size distributions show a reduction in the major pore diameter from 14.2 to 11 Å (see Supporting Information, Section S7), suggesting that the transformation from alkyne to dibromoalkene units results in a mechanical contraction of the ligand and thus the MOF.

Single crystals of both **4** and **5** were suspended in CHCl_3_ and exposed to Br_2_ for four days, thereafter the CHCl_3_ was replenished several times before finally resolvating in DMF (see Supporting Information, Section S9). Both MOFs were successfully brominated in a single‐crystal to single‐crystal manner with data collections possible, resulting in an accurate description of the structural alterations introduced during bromination. The brominated MOFs were found to retain the same *Fd* $\bar{3}$ *m* space group as their parent structures, with bromination resulting in a mechanical contraction of the MOFs, as observed through reductions in the unit cell edges from 39.8116(7) Å to 39.067(7) Å as **4** is brominated to **4‐Br_4_** and from 39.806(5) Å to 39.0451(3) Å as **5** is brominated to **5‐Br_4_**. The solid‐state structures of **4‐Br_4_** and **5‐Br_4_** exhibit significant disorder in their linker components, as would be expected due to the number of frustrated positions the dissymmetric linker could adopt along the linear vector.

**Figure 4:**
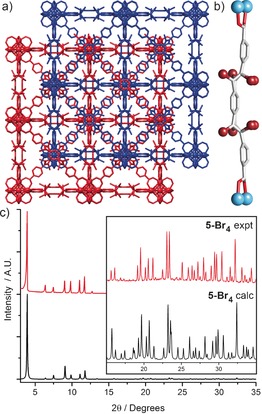
a) Representation of the crystal structure of **5‐Br_4_**, with the two interpenetrated nets coloured red and blue. Disorder in the peb‐Br_4_^2−^ linker has not been removed. b) Despite the disorder, it is clear that peb‐Br_4_^2−^ is found in the *trans*,*trans* configuration, although rotation of the central benzene ring results in multiple bromine positions. c) The disorder model proposed for **5‐Br_4_** is validated by the correlation between the PXRD pattern predicted from the single crystal structure and the experimental pattern. Atom colouring: Hf (pale blue); C (grey); O (red); Br (brown). H atoms are omitted for clarity.

In the crystal structure (Figure 4 a) of **5‐Br_4_**, it is possible, despite the linker disorder resulting in multiple bromine positions, to visualise the *trans*,*trans*‐configuration of the peb‐Br_4_^2−^ linker (Figure 4 b). The contraction on bromination brings the two interpenetrated nets closer together, narrowing the pores as can be seen in the N_2_ adsorption isotherms (Figure 3 f). The disorder model of **5‐Br_4_**, as well as that of the more disordered **4‐Br_4_** (see Supporting Information, Section S9), indicates that the central benzene ring of the linker is free to rotate. Comparison of the experimental PXRD pattern with the pattern calculated from our structure model of **5‐Br_4_** shows good agreement (Figure 4 c), suggesting that the disorder model is valid and that bromination occurs quantitatively to produce phase‐pure material.

**Figure 5:**
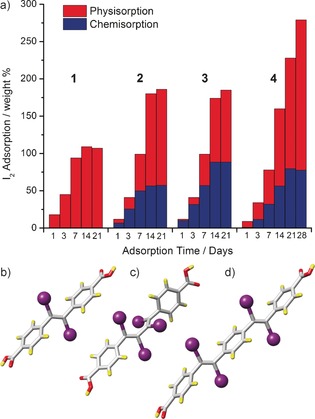
a) Summary of the physisorption and chemisorption I_2_ storage capacities of **1**–**4**. Representations of the solid‐state structures of b) *trans*‐edb‐I_2_‐H_2_, c) *trans*‐*trans*‐bdb‐I_4_‐H_2_, and d) *trans*‐*trans*‐peb‐I_4_‐H_2_, isolated from digested samples of **2**–**4**, respectively, after iodination. Atom colouring: C (grey); O (red); H (yellow); I (purple).

The facile addition of bromine across the integral functional units of **1**–**4** prompted us to consider the irreversible trapping of harmful radioactive iodine released from the nuclear fission of uranium in nuclear processing applications.^36^ Several porous materials have been investigated for physisorption of radioactive iodine, including MOFs (e.g. ZIFs,^37^ HKUST‐1^38^), although charged porous aromatic frameworks (PAFs) have recently been shown to be promising candidates with PAF‐24 able to capture 276 % iodine by weight.^39^ To investigate the iodine storage capacities of the Zr MOFs **1**–**4**, microcrystalline samples were exposed to iodine vapours (see Supporting Information, Section S10), with the total iodine uptakes measured through gravimetric investigations, alongside ^1^H NMR spectroscopic analysis to establish the percentage chemisorption (Figure 5 a).

Surprisingly we find that **1** is unable to chemisorb I_2_ across its integral alkene units, despite both bromination and bromohydrination occurring in a facile manner in solution; however, a maximum I_2_ physisorption storage capacity of 107 % *w*/*w* was recorded. Both **2** and **3** demonstrate much larger total I_2_ storage capacities as they can both chemi‐ and physisorb I_2_, and the presence of twice as many alkyne units in **3** is reflected in the chemisorption capacities, with **2** demonstrating a maximum of 57 % *w*/*w* irreversible trapping of I_2_, compared to 88.5 % *w*/*w* for **3**. The interpenetrated material **4** shows the highest storage value, with a maximum uptake of 279 % *w*/*w* recorded, comparable to the recent benchmark capacity of 276 % *w*/*w* set by PAF‐24.^39^ The I_2_ chemisorption capacity of **4** is lower than that of **3**, even though both materials contain a similar alkyne content; hence, the superior uptake is the result of a high tendency of I_2_ to physisorb within the pores, possibly due to the high density of Zr_6_ clusters as a result of interpenetration. Chemisorption by **3** may also lead to partial pore collapse, as the analogous **3‐Br_4_** is not porous, thus hindering later I_2_ uptake.

The stereoselectivities of these vapour phase iodinations are analogous to the solution phase brominations, as can be seen by the ^1^H NMR spectra of digested samples used to monitor the extent of chemisorption. Additionally, crystals of iodinated linkers separated from a number of the [D_6_]DMSO/D_2_SO_4_ digest solutions (see Supporting Information, Section S11), allowing us to crystallographically characterise *trans*‐edb‐I_2_‐H_2_,23a *trans*,*trans*‐bdb‐I_4_‐H_2_ and *trans*,*trans*‐peb‐I_4_‐H_2_, from the iodination of **2**, **3** and **4**, in turn (Figure 5 b‐5d). These results unambiguously show that the chemical stability of Zr MOFs, in concert with reactive chemisorptive sites on the linkers, generates potential candidates for I_2_ storage/capture applications. Additionally, proof‐of‐concept experiments show that chemisorbed halogens can be abstracted by pyrrolidine to regenerate the parent MOF; **1‐Br_2_** can be completely converted back to **1** by soaking in an acetone solution with 15 equivalents of pyrrolidine for 42 h (see Supporting Information, Section S12).

## Conclusion

In conclusion, we have demonstrated that the combined chemical and mechanical stabilities of Zr and Hf MOFs enable their postsynthetic halogenation across integral unsaturated linker functionalities. Both solution‐phase bromination and vapour‐phase iodination proceed stereoselectively and in excellent yields across alkenes, alkynes, butadiynes and in interpenetrated frameworks, while the water stability of the Zr MOFs allows aqueous bromohydrination of alkene units. Bromination is shown to be reversible by abstraction with pyrrolidine to restore the parent material, making it an unusual example of reversible covalent postsynthetic modification, and we are currently investigating the potential for multiple cycles of sequestration and regeneration of the MOFs. The chemisorption of I_2_ vapours by the MOFs, combined with their high porosity, makes them excellent candidates for sequestration of radioactive I_2_, and we are currently examining the scope of substrates which can be irreversibly captured by the MOFs in this manner. In addition, the change in hybridisation of integral linker atoms upon halogenation will modulate the mechanical properties of the MOFs, and we are investigating these effects in detail.

## Experimental Section

Experimental details for this work can be found in the Supporting Information. CCDC 1443195–1443201 contain the supplementary crystallographic data. These data can be obtained free of charge by The Cambridge Crystallographic Data Centre.

## Supporting information

As a service to our authors and readers, this journal provides supporting information supplied by the authors. Such materials are peer reviewed and may be re‐organized for online delivery, but are not copy‐edited or typeset. Technical support issues arising from supporting information (other than missing files) should be addressed to the authors.

Supplementary
