# Supplementary material for: Stereoselective Halogenation of Integral Unsaturated C‐C Bonds in Chemically and Mechanically Robust Zr and Hf MOFs
Source: Chemistry. 2016 Feb 24;22(14):4870–7. doi: 10.1002/chem.201505185 (PMC5067641; doi:10.1002/chem.201505185)
Supplement: Supplementary file 1 — Supplementary [file CHEM-22-4870-s001.pdf]

CHEMISTRY
A European Journal

Supporting Information

Stereoselective Halogenation of Integral Unsaturated C-C Bonds in Chemically and Mechanically Robust Zr and Hf MOFs

Ross J. Marshall, Sarah L. Griffin, Claire Wilson, and Ross S. Forgan*[a]

chem_201505185_sm_miscellaneous_information.pdf

SUPPORTING INFORMATION

Table of Contents:

**S1.**	**General Experimental Remarks.**	**S2.**
**S2.**	**Ligand Synthesis.**	**S4.**
**S3.**	**MOF Synthesis.**	**S8.**
**S4.**	**Alternative Bromination of (1).**	**S14.**
**S5.**	**Bulk Postsynthetic Bromination of (3).**	**S18.**
**S6.**	**Solution Phase Bromination of bdb-Me$_2$.**	**S22.**
**S7.**	**Bulk Postsynthetic Bromination of (4) and (5).**	**S24.**
**S8.**	**Solution Phase Bromination of peb-Me$_2$.**	**S31.**
**S9.**	**Single-Crystal to Single-Crystal Bromination of (4) and (5).**	**S33.**
**S10.**	**Iodination of (1), (2), (3) and (4).**	**S36.**
**S11.**	**Crystal Structures of Iodinated Linkers.**	**S44.**
**S12.**	**Dehalogenation of (1-Br$_2$).**	**S46.**
**S13.**	**References.**	**S48.**

S1. General Experimental Remarks

All chemicals and solvents were purchased from Alfa Aesar, Fisher Scientific, Fluorochem, Merck Millipore, Sigma-Aldrich, Strem Chemicals and VWR and used without further purification.

Powder X-ray Diffraction (PXRD): PXRD measurements were carried out at 298 K using a PANalytical X'Pert PRO diffractometer (λ (CuKα) = 1.4505 Å) on a mounted bracket sample stage. Data were collected over the range 3–45 °. PXRD patterns were predicted from single crystal data using Mercury 3.5.1.[S1] (University of Glasgow)

Single Crystal Diffraction (SCXRD): Data for **(3)**, **(3-Br$_4$)**, **(4)**, **(4-Br$_4$)** and **(5-Br$_4$)** were collected using a Rigaku AFC12 goniometer equipped with an enhanced sensitivity (HG) Saturn724+ detector mounted at the window of an FR-E+ SuperBright molybdenum rotating anode generator with VHF Varimax optics (70 µm focus) equipped with an Oxford Cryosystems cryostream device. (EPSRC UK National Crystallography Service)

Data for **(5)** and *trans*,*trans*-peb-I_4-H_2 were collected using a Bruker ApexII CCD kappa goniometer with a Mo sealed tube source and equipped with an Oxford Cryosystems n-Helix device. Data for *trans*,*trans*-bdb-I_4-H_2 were collected using a Nonius KappaCCD with a Mo sealed tube source and equipped with an Oxford Cryosystems cryostream device. (University of Glasgow)

Bromine Analysis: Carried out by MEDAC Ltd, analytical and chemical consultancy services, Surrey, UK.

Thermal Gravimetric Analysis (TGA): Measurements were carried out using a TA Instruments Q500 Thermogravimetric Analyser. Measurements were collected from room temperature to 1000 °C with a heating rate of 10 °C / min under an air atmosphere. (University of Glasgow)

Nuclear Magnetic Resonance (NMR): NMR spectra were recorded on either a Bruker AVIII 400 MHz spectrometer or a Bruker AVI 500 MHz Spectrometer and referenced to residual solvent peaks unless stated otherwise. (University of Glasgow)

Raman Spectroscopy: Raman spectra were collected on a LabRAM HR system using a Ventus 532 laser system (λ = 532 nm, 100 mW), equipped with a Synapse CCD detection system. The brominated materials were sensitive to the Raman laser (λ = 532 nm, 100 mW), and so the spectra have lower resolution than those of the parent materials. (University of Glasgow)

Gas Uptake: N_2 adsorption isotherms were carried out at 77 K on a Quantachrome Autosorb iQ gas sorption analyser. Samples were degassed under vacuum at 120 °C for 20 h using the internal turbo pump. BET surface areas were calculated from the isotherms using the Micropore BET Assistant in the Quantachrome ASiQwin operating software. (University of Glasgow)

Molecular Dynamics: Minimisation of bdb-Br_4-H_4 was carried out using the UFF forcefield in a steepest descent, four step update protocol, using the auto-optimisation tool in Avogadro 1.1.1 software suite, with a planar structure imported from Chemdraw Prime version 15 as an input file. (University of Glasgow)

S2. Ligand Synthesis

The chemical structures and abbreviated names of all ligands used in this study – generated postsynthetically or otherwise – are detailed in Scheme S1.

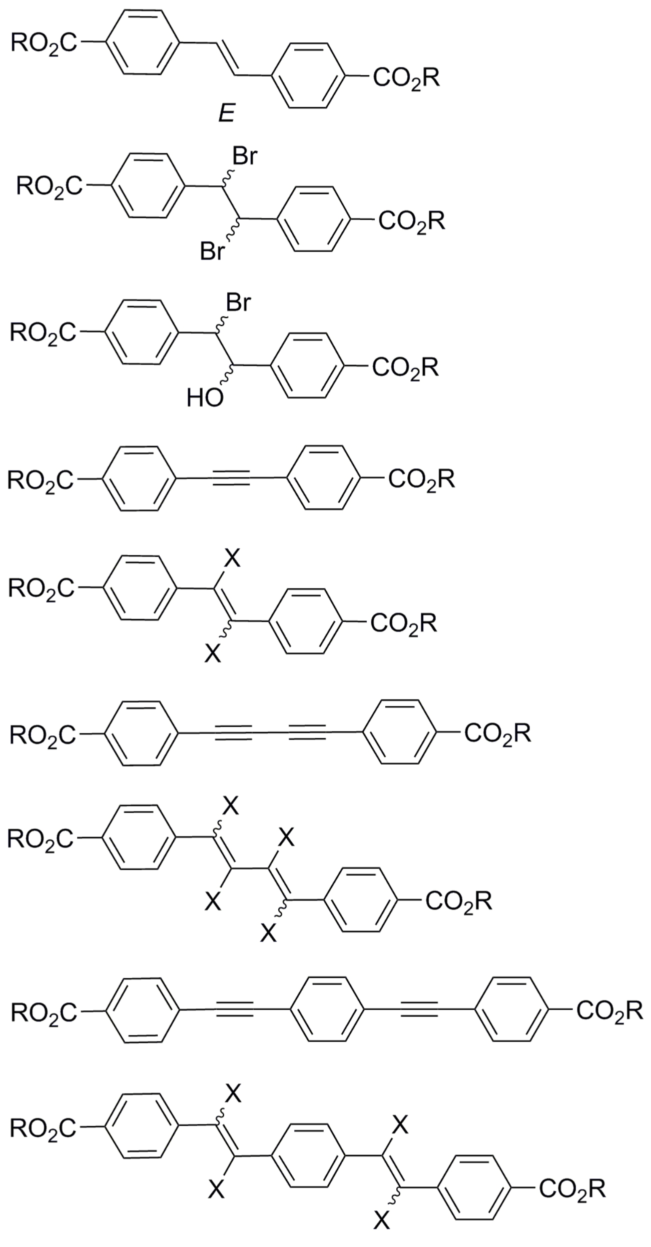

R	Compound	Isomerism
CH_3	sdc-Me_2	n/a
H	sdc-H_2	
CH_3	sdc-Br_2-Me_2	*(R,R)/(S,S)/meso*
H	sdc-Br_2-H_2	
CH_3	sdc-Br-OH-Me_2	*(R,R)/(S,S)/(R,S)/(S,R)*
H	sdc-Br-OH-H_2	
CH_3	edb-Me_2	n/a
H	edb-H_2	
CH_3	edb-X_2-Me_2	*cis/trans*
H	edb-X_2-H_2	
CH_3	bdb-Me_2	n/a
H	bdb-H_2	
CH_3	bdb-X_4-Me_2	*trans,trans/trans,cis/cis,cis*
H	bdb-X_4-H_2	
CH_3	peb-Me_2	n/a
H	peb-H_2	
CH_3	peb-X_4-Me_2	*trans,trans/trans,cis/cis,cis*
H	peb-X_4-H_2	

Scheme S1. Chemical structures of compounds and the abbreviation scheme used in this study (X= I or Br).

The alkyne-bridged ligand 4,4′-ethynylenedibenzoic acid (edb-H_2) was prepared as described previously from its dimethyl ester.[S2] The precursor methyl 4-ethynylbenzoate, required for the synthesis of bdb-Me_2 and peb-Me_2, was obtained via deprotection of methyl 4-(2-(trimethylsilyl)ethynyl)benzoate.[S3] If no ^{13}C NMR spectroscopic data are provided, it is because the compound was not sufficiently soluble to obtain a spectrum.

Methyl 4-(2-(trimethylsilyl)ethynyl)benzoate

Methyl 4-iodobenzoate (11.400 g, 43.50 mmol) and trimethylsilylacetylene (6.80 ml, 47.77 mmol) were added to a stirring solution of THF (80 ml) and TEA (20 ml). The solution was degassed for 10 minutes before bis(triphenylphosphine)palladium(II) dichloride (0.914 g, 1.30 mmol) and copper(I) iodide (0.496 g, 2.60 mmol) were added under an N_2 atmosphere. The reaction mixture was subject to reflux at 80 °C for 4 hours under an N_2 atmosphere. After cooling to room temperature, the resulting mixture was filtered over celite and washed with diethyl ether until washings were colourless. The organic phase was washed with saturated NH_4Cl (aq) (2 x 100 ml), brine (1 x 100 ml), dried over $MgSO_4$ and the filtrate collected. The solvent was removed under reduced pressure to yield a dark solid. The product was purified by flash chromatography eluting hexane:DCM (2:1), yielding the product as a light coloured solid (8.508 g, 36.60 mmol, 84%). ^{1}H NMR ($CDCl_3$): δ/ppm 0.26 (s, 9H), 3.91 (s, 3H), 7.52 (d, 2H, J = 8.6 Hz), 7.97 (d, 2H, J = 8.6 Hz); ^{13}C NMR ($CDCl_3$): δ/ppm 0.0 (CH_3), 52.4 (CH_3), 97.9 (C), 104.2 (C), 128.0 (C), 129.5 (CH), 129.9 (C), 132.0 (CH), 166.7 (C); HRMS (CI) calculated for $C_{13}H_{17}O_2Si$ $(M+H)^+$ 232.0998, found *m/z* 232.0995.

Methyl 4-ethynylbenzoate

Methyl 4-(2-(trimethylsilyl)ethynyl)benzoate (8.508 g, 36.62 mmol, 1 eq) was dissolved in a solution of DCM (200 ml) and MeOH (20 ml) by stirring. Potassium carbonate (10.117 g, 73.20 mmol, 2 eq) was added and the mixture was stirred at room temperature for 3 hours. A colourless filtrate was collected by eluting through a silica plug with MeOH. The solvent was removed under reduced pressure to yield an off white solid. The solid was re-dissolved in $CHCl_3$ and filtered to remove any residual silica, which was then removed under reduced pressure to yield the final product (5.237 g, 32.69 mmol, 89%). ^{1}H NMR ($CDCl_3$): δ/ppm 3.23 (s, 1H), 3.92 (s, 3H), 7.55 (d, 2H, J = 8.7 Hz), 8.00 (d, 2H, J = 8.7 Hz); ^{13}C NMR ($CDCl_3$): δ/ppm 52.4 (CH_3), 80.2 (CH), 82.9 (C), 126.9 (C), 129.6 (CH), 130.3 (C), 132.2 (CH), 166.6 (C); HRMS (CI) calculated for $C_{10}H_8O_2$ $(M)^+$ 160.0524, found *m/z* 160.0522.

The compound dimethyl 4,4′-(buta-1,3-diyne-1,4-diyl)dibenzoate (bdb-Me_2) was synthesised according to modified literature procedures[S4] with subsequent saponification resulting in the 4,4′-(buta-1,3-diyne-1,4-diyl)dibenzoic acid ligand (bdb-H_2).[S2]

bdb-Me_2

Methyl 4-ethynylbenzoate (0.641 g, 4.00 mmol), tetramethylethylenediamine (0.60 ml, 4.01 mmol) and DMF (20 ml) were added to a round bottom flask under an N_2 atmosphere. Copper(II) chloride (0.268 g, 1.99 mmol) was added and the reaction mixture was left to stir at 40 °C under an N_2 atmosphere. After 2 hours, the reaction was quenched by addition of EtOAc. The solvent was removed under reduced pressure, yielding a light green solid. The product was dissolved in $CHCl_3$ and washed several times with an aqueous solution of ethylenediaminetetraacetic acid disodium salt dihydrate until a light yellow solution remained. The organic phase was then washed with brine (2 x 40 ml), dried over $MgSO_4$ and filtered. The filtrate was collected and the solvent removed under reduced pressure to yield a light yellow solid (0.498 g, 1.56 mmol, 78%). ^{1}H NMR ($CDCl_3$): δ/ppm 3.93 (s, 6H), 7.59 (d, 4H, J = 8.7 Hz), 8.02 (d, 4H, J = 8.7 Hz); ^{13}C NMR ($CDCl_3$): δ/ppm 52.4 (CH_3), 76.3 (C), 81.8 (C), 126.1 (C), 129.6 (CH), 130.6 (C), 132.5 (CH), 166.3 (C); HRMS (CI) calculated for $C_{20}H_{14}O_4$ $(M)^+$ 318.0892, found m/z 318.0895.

bdb-H_2

bdb-Me_2 (0.650 g, 2.04 mmol, 1 eq) was suspended in EtOH (40 ml) with stirring. Potassium hydroxide (0.286 g, 5.10 mmol, 2.5 eq) was dissolved separately in H_2O (40 ml) then added to the flask containing the diester and subject to reflux overnight. The transparent solution was then removed from the heat and allowed to cool to room temperature, before the product was precipitated by addition of 6 M HCl (aq). The product was collected by centrifugation multiple times with H_2O (40 ml) until washings were neutral. The light coloured product was dried under vacuum (0.469 g, 1.62 mmol, 79%). ^{1}H NMR (DMSO-d_6): δ/ppm 7.75 (d, 4H, J = 8.6 Hz), 7.98 (d, 4H, J = 8.6 Hz); HRMS (ESI) calculated for $C_{18}H_9O_4$ $(M\text{-}H)^-$ 289.0506, found m/z 289.0510.

The compound dimethyl 4,4′-[1,4-phenylenebis(ethyne-2,1-diyl)]-dibenzoate (peb-Me_2) was synthesised according to modified literature procedures[S5] with subsequent saponification resulting in the 4,4′-[1,4-phenylenebis(ethyne-2,1-diyl)]-dibenzoic acid ligand (peb-H_2).[S6]

peb-Me_2

Methyl 4-ethynylbenzoate (2.497 g, 15.59 mmol, 2.5 eq) was dissolved in TEA (160 ml) by stirring. The solution was degassed for 10 minutes, before 1,4-diiodobenzene (2.059 g. 6.24 mmol, 1 eq), bis(triphenylphosphine)palladium(II) dichloride (0.295g, 0.42 mmol) and copper(I) iodide (0.072 g, 0.38 mmol) were added. The reaction mixture was left to stir at 50 °C overnight under an N_2 atmosphere. The mixture was removed from the heat and once cooled to room temperature the product was collected by vacuum filtration and washed with hexane until washings were colourless. The product was stirred overnight in H_2O (300 ml), then collected by vacuum filtration. The product was stirred in a minimal volume of DCM, collected by vacuum filtration and dried under vacuum (2.445 g, 6.20 mmol, 99%). ^1H NMR ($CDCl_3$): δ/ppm 3.94 (s, 6H), 7.54 (s, 4H), 7.60 (d, 4H, J = 8.6 Hz), 8.04 (d, 4H, J = 8.6 Hz); HRMS (ESI) calculated for $C_{26}H_{18}NaO_4$ $(M+Na)^+$ 417.1103, found *m/z* 417.1052.

peb-H_2

peb-Me_2 (1.892 g, 4.80 mmol, 1 eq) was dissolved in 1:1 MeOH/THF (350 ml). Potassium hydroxide (2.693 g, 48.00 mmol, 10 eq) was dissolved separately in H_2O, and added to the solution containing the diester. The resulting solution was allowed to reflux overnight at 85 °C. The transparent solution was then removed from the heat and allowed to cool to room temperature, before the product was precipitated by addition of 6 M HCl (aq). The product was collected by vacuum filtration and washed with copious amounts of water until neutral. The light coloured solid was dried under vacuum (0.931 g, 2.54 mmol, 53%). ^1H NMR (DMSO-d_6): δ/ppm 7.67 (s, 4H), 7.70 (d, 4H, J = 8.6 Hz), 8.0 (d, 4H, J = 8.6 Hz); HRMS (ESI) calculated for $C_{24}H_{12}O_4Na$ $(M-2H+Na)^-$ 387.0639, found *m/z* 387.0620.

S3. MOF Synthesis

The synthesis of MOFs containing the previously described ligands (Section S2) is detailed in this section. The MOFs have been assigned numbers for simplification (Scheme S2) and this classification is used throughout the remainder of this study, with postsynthetically derived materials adopting their parent's number followed by an indication of the modification performed, e.g. **(1-Br$_2$)** corresponds to the postsynthetically brominated Zr MOF **(1)**.

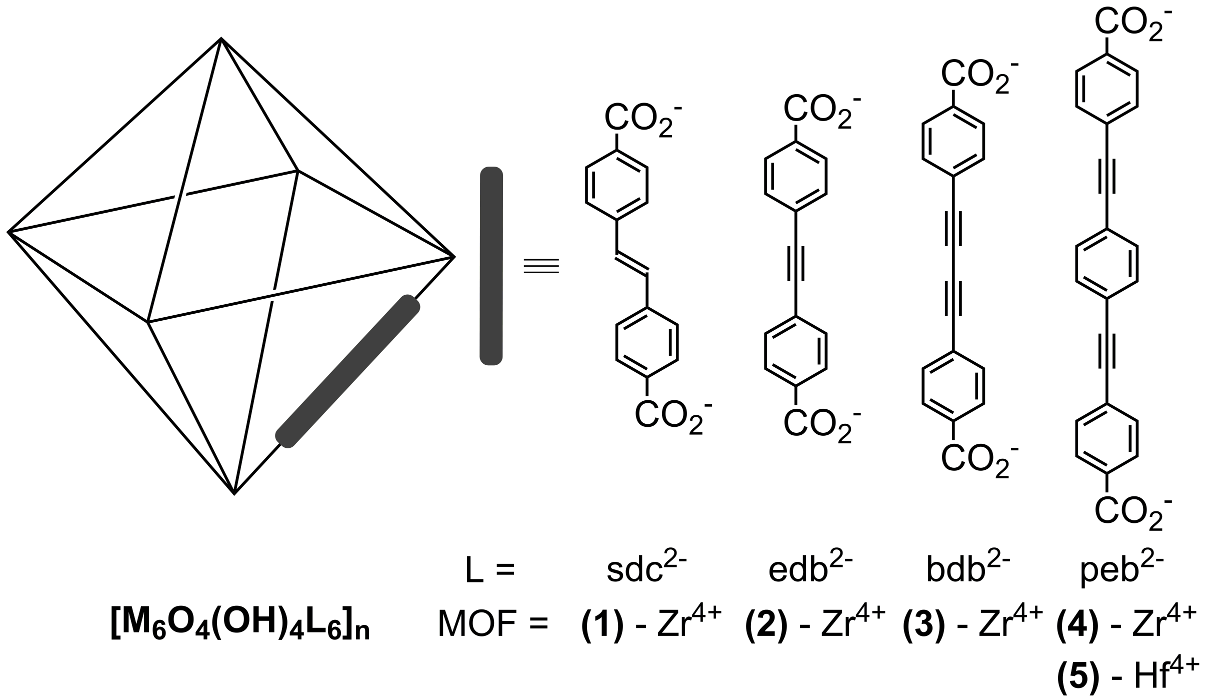

Scheme S2. Abbreviation scheme of the ligands and MOFs used throughout this study.

Both bulk and single crystal samples of $[Zr_6O_4(OH)_4(sdc)_6]_n$ **(1)**, $[Zr_6O_4(OH)_4(\textit{meso}\text{-sdc-}Br_2)_6]_n$ **(1-Br$_2$)**, $[Zr_6O_4(OH)_4(edb)_6]_n$ **(2)**, and $[Zr_6O_4(OH)_4(\textit{trans}\text{-edb-}Br_2)_6]_n$ **(2-Br$_2$)** have been previously reported by our group, with the parent MOFs synthesised according to our amino acid modulated protocol.[S7, S8] The syntheses of $[Zr_6O_4(OH)_4(bdb)_6]_n$ **(3)**, $[Zr_6O_4(OH)_4(bdb\text{-}Br_4)_6]_n$ **(3-Br$_4$)**, $[Zr_6O_4(OH)_4(peb)_6]_n$ **(4)**, $[Zr_6O_4(OH)_4(peb\text{-}Br_4)_6]_n$ **(4-Br$_4$)**, $[Hf_6O_4(OH)_4(peb)_6]_n$ **(5)**, and $[Hf_6O_4(OH)_4(peb\text{-}Br_4)_6]_n$ **(5-Br$_4$)** are carried out using similar protocols described herein, although benzoic acid modulation was employed in some cases.[S9]

Bulk Material **(3)**

Benzoic acid (1.649 g, 13.50 mmol, 30 eq), zirconium chloride (0.105 g, 0.45 mmol, 1 eq) and DMF (20 ml) were added to a 50 ml PYREX reagent bottle and sonicated for 10 minutes. bdb-H_2 (0.131 g, 0.45 mmol, 1 eq) and concentrated HCl (0.04 ml) were added and the suspension was sonicated for a further 10 minutes, before being placed in the oven at 120 °C for 72 hours. The bulk material was collected from the bottle upon completion, centrifuged once with fresh DMF (30 ml) and twice with acetone (2 x 30 ml), before being dried under vacuum ($[Zr_6O_4(OH)_4(bdb)_6]_n$, 0.102 g, 0.04 mmol, 53% - average yield over 4 reactions). Bromine analysis: 0% calculated; 1.21% found.

Activation: Powder samples were added to 50 ml PYREX reagent bottles and left to stand in $CHCl_3$. The $CHCl_3$ was exchanged for fresh $CHCl_3$ a further 4 times over 4 days, before being collected by centrifugation and dried under vacuum.

Single Crystals **(3)**

Single crystals of **(3)** were synthesised in the presence of both L-proline and benzoic acid as modulator. We found that bulk microcrystalline samples of **(3)** prepared using benzoic acid as modulator exhibited superior porosity, and so benzoic acid was used throughout for experiments involving single crystals or bulk samples of **(3)**.

Benzoic acid (0.824 g, 6.75 mmol, 30 eq), zirconium chloride (0.052 g, 0.22 mmol, 1 eq) and DMF (13 ml) were added to a 50 ml PYREX reagent bottle and sonicated for 10 minutes. bdb-H_2 (0.065 g, 0.22 mmol, 1 eq) and concentrated HCl (0.02 ml) were added and the suspension was sonicated for a further 10 minutes before being placed in the oven at 120 °C for 24 hours. The bottles were removed from the oven after this period, and allowed to cool to room temperature. The crystals were left to stand in their mother solution.

Single crystal X-ray diffraction data were collected using CrystalClear-SM Expert 3.1 b27[S10] and data processing carried out using CrysAlis PRO 1.171.38.41.[S11] The structure was solved using SHELXS and the structure refined using SHELXL[S12] within Olex2.[S13] Anisotropic adps were refined for Zr and O atoms only, similarity restraints were applied to the C atom Uiso values, and distance restraints were applied to all C-C distances. Hydrogen atoms were placed in geometrically calculated positions and included as part of a riding model. The OH hydrogens are not included explicitly in the model but are included in the

formula and all values derived from the unit cell contents. Almost 80% of the cell volume is not occupied by the framework and contains diffuse and disordered solvent molecules. This electron density was accounted for using SQUEEZE within PLATON[S14] which calculated a solvent accessible volume of 30003 Å^3, containing 3394 electrons, equivalent to approximately 85 molecules of DMF. The high final R-factors are thought to be a consequence of the high level of disorder in the material. The structure was deposited with the Cambridge Structural Database with deposition number CCDC 1443195.

Crystal data for **(3)**. $C_{108}H_{52}O_{32}Zr_6$, M_r = 2408.81, Cubic, a = 33.3694 (3) Å, V = 37157.4 (10) Å^3, T = 100 K, space group Fm-3m (no. 225), Z = 4, 49850 measured reflections, 1676 unique (R_{int} = 0.058), which were used in all calculations. The final R_1 = 0.219 for 1424 observed data $R[F^2 > 2\sigma(F^2)]$ and $wR(F^2)$ = 0.605 (all data).

Bulk Material **(4)**

L-proline (0.130 g, 1.13 mmol, 5 eq), zirconium chloride (0.052 g, 0.22 mmol, 1 eq) and DMF (10 ml) were added to a 50 ml PYREX reagent bottle and sonicated for 10 minutes. peb-H_2 (0.082 g, 0.22 mmol, 1 eq) and concentrated HCl (0.02 ml) were added and the suspension was sonicated for a further 10 minutes before being placed in the oven at 120 °C for 60 hours. The bulk material was collected from the bottle upon completion, centrifuged once with fresh DMF (30 ml) and twice with acetone (2 x 30 ml), before being dried under vacuum ($[Zr_6O_4(OH)_4(peb)_6]_n$, 0.099 g, 0.035 mmol, 95% - average yield over 2 reactions). Bromine analysis: 0% calculated; 0.6% found.

Activation: Powder samples were added to 50 ml PYREX reagent bottles and left to stand in $CHCl_3$. The $CHCl_3$ was exchanged for fresh $CHCl_3$ a further 4 times over 4 days, before being collected by centrifugation and dried under vacuum.

Single Crystals **(4)**

Single crystal synthesis of **(4)** was attempted using either L-proline or benzoic acid modulation, as in our experience these two modulators have provided the greatest enhancement of single crystal growth of Zr/Hf MOFs constructed from linear dicarboxylic acid ligands.[S7, S8] However, upon visual inspection of the crystals obtained from both sets of synthetic conditions it was obvious that the crystals obtained in the presence of benzoic acid

were much more defined in shape and were also larger in size (~100 µm compared with ~50 µm) than those synthesised using L-proline modulation. This difference was reflected during single crystal X-ray diffraction, with only poor low resolution data obtained for the L-proline modulated crystals. Therefore, single crystals were synthesised in the presence of benzoic acid to aid single crystal X-ray data collections (similar results were observed for the Hf analogue **(5)**).

Benzoic acid (0.824 g, 6.75 mmol, 30 eq), zirconium chloride (0.052 g, 0.22 mmol, 1 eq) and DMF (13 ml) were added to a 50 ml PYREX reagent bottle and sonicated for 10 minutes. peb-H_2 (0.082 g, 0.22 mmol, 1 eq) and HCl (0.02 ml) were added and the suspension was sonicated for a further 10 minutes before being placed in the oven at 120 °C for 24 hours. The bottles were removed from the oven after this period, and allowed to cool to room temperature. The crystals were left to stand in their mother solution.

Single crystal diffraction data were collected and processed using CrystalClear-SM Expert 3.1 b27.[S10] The structure was solved using Superflip[S15] and refined using SHELXL[S12] within Olex2.[S13] All non-hydrogen atoms were refined with anisotropic atomic displacement parameters (adps) with an enhanced rigid-body restraint (RIGU) applied. Hydrogen atoms were placed in geometrically calculated positions and included as part of a riding model, except the OH hydrogen atoms, which were not included explicitly in the model but are included in the cell contents and all values derived from them. Approximately 63% of the cell volume is not occupied by the framework and contains diffuse and disordered solvent molecules. This electron density was accounted for using SQUEEZE within PLATON[S14] which calculated a solvent accessible volume of 40049 Å^3 containing 7636 electrons, equivalent to approximately 190 molecules of DMF. The structure was deposited with the Cambridge Structural Database with deposition number CCDC 1443196.

Crystal data for **(4)**. $C_{144}H_{76}O_{32}Zr_6$, M_r = 2865.36, Cubic, a = 39.8116 (7) Å, V = 63100 (3) Å^3, T = 100 K, space group Fd-3m (no. 227), Z = 8, 85487 measured reflections, 3407 unique (R_{int} = 0.040), which were used in all calculations. The final R_1 = 0.082 for 3404 observed data $R[F^2 > 2\sigma(F^2)]$ and $wR(F^2)$ = 0.211 (all data).

Bulk Material **(5)**

L-proline (0.130 g, 1.13 mmol, 5 eq), hafnium chloride (0.072 g, 0.22 mmol, 1 eq) and DMF (10 ml) were added to a 50 ml PYREX reagent bottle and sonicated for 10 minutes. peb-H_2 (0.082 g, 0.22 mmol, 1 eq) and HCl (0.02 ml) were added and the suspension was sonicated for a further 10 minutes before being placed in the oven at 120 °C for 60 hours. The bulk material was collected from the bottle upon completion, centrifuged once with fresh DMF (30 ml) and twice with acetone (2 x 30 ml), before being dried under vacuum ($[Hf_6O_4(OH)_4(peb)_6]_n$, 0.120 g, 0.035 mmol, 95% - average yield over 2 reactions). Bromine analysis: 0% calculated; 0.1% found.

Activation: Powder samples were added to 50 ml PYREX reagent bottles and left to stand in $CHCl_3$. The $CHCl_3$ was exchanged for fresh $CHCl_3$ a further 4 times over 4 days, before being collected by centrifugation and dried under vacuum.

Single Crystals **(5)**

Benzoic acid (0.824 g, 6.75 mmol, 30 eq), hafnium chloride (0.072 g, 0.22 mmol, 1 eq) and DMF (13 ml) were added to a 50 ml PYREX reagent bottle and sonicated for 10 minutes. peb-H_2 (0.082 g, 0.22 mmol, 1 eq) and HCl (0.02 ml) were added and the suspension was sonicated for a further 10 minutes before being placed in the oven at 120 °C for 24 hours. The bottles were removed from the oven after this period, and allowed to cool to room temperature. The crystals were left to stand in their mother solution.

Single crystal X-ray diffraction data were collected and processed using APEX2,[S16] the structure was solved using Superflip[S15] and refined using SHELXL[S12] within Olex2.[S13] All non-hydrogen atoms were refined with anisotropic atomic displacement parameters (adps) with an enhanced rigid-body restraint (RIGU) and similarity restraint applied to the adps for C1 and an ISOR restraint to C1 and O3. Hydrogen atoms were placed in geometrically calculated positions and included as part of a riding model, except the OH hydrogen atoms, which were not included explicitly in the model but are included in the cell contents and all values derived from them. Approximately 63% of the cell volume is not occupied by the framework and contains diffuse and disordered solvent molecules. This electron density was accounted for using SQUEEZE within PLATON[S14] which calculated a solvent accessible volume of 40068 Å^3 containing 19296 electrons. The structure was deposited with the Cambridge Structural Database with deposition number CCDC 1443197.

Crystal data for **(5)**. $C_{144}H_{76}Hf_6O_{32}$, M_r = 3388.98, Cubic, a = 39.806 (5) Å, V = 63074 (25) Å^3, T = 100 K, space group Fd-3m (no. 227), Z = 8, 30725 measured reflections, 2646 unique (R_{int} = 0.225), which were used in all calculations. The final R_1 = 0.109 for 1830 observed data $R[F^2 > 2\sigma(F^2)]$ and $wR(F^2)$ = 0.199 (all data).

S4. Alternative Bromination of (1)

We have recently reported the successful bromination of $[Zr_6O_4(OH)_4(sdc)_6]_n$ **(1)** to $[Zr_6O_4(OH)_4($*meso*-sdc-$Br_2)_6]_n$ **(1-Br₂)**, both on bulk microcrystalline and single crystal samples, using neat bromine as the brominating agent.[S7] The high chemical stability of **(1)** enables the postsynthetic bromination to occur quantitatively whilst retaining crystallinity. However, we are aware that many of the MOFs desirable for these modifications may not be as chemically or mechanically stable as zirconium MOFs, and as such we have decided to investigate the use of *N*-bromosuccinimide (NBS) in the presence of *N,N'*-diphenylthiourea (DPT) as a milder, alternative brominating agent.[S17] The use of NBS in the presence of H_2O is known to result in bromohydrination and so this was also explored as a potential route for the functionalisation of **(1)** (Scheme S3).

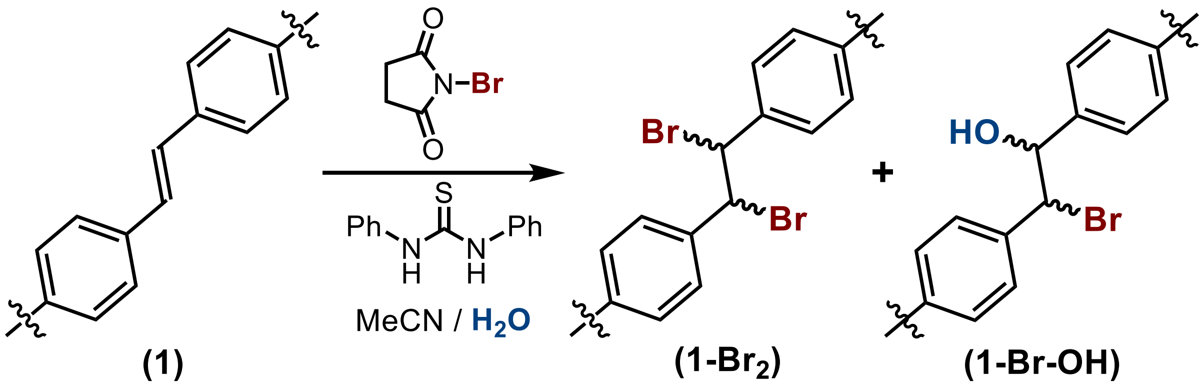

Scheme S3. Reaction scheme for postsynthetic modification of **(1)** with NBS, highlighting potential products and the abbreviations used.

<u>General Procedure for Postsynthetic Modification of (1) with NBS</u>

(1) (0.020 g, 0.053 mmol alkene, 1 eq) was suspended in the reaction solvent (5 ml) by stirring, then *N,N'*-diphenylthiourea (DPT) (10 mol %) was added if required, before cooling to 0 °C. *N*-Bromosuccinimide (0.047 g, 0.265 mmol, 5 eq) was added, the flask was sealed and the mixture was purged by passing N_2 through the system. The reaction mixture was left to stir under an N_2 atmosphere overnight, gradually warming to room temperature. The modified MOF was collected by centrifugation with fresh acetonitrile (2 x 10 ml) and acetone (2 x 10 ml), before being dried under vacuum.

^1H NMR spectroscopy of acid digested samples (DMSO-d_6/D_2SO_4) of the resulting MOFs was used to investigate the effect of reaction conditions on product distribution (Table S1).

Table S1. Summary of reaction conditions investigated during the postsynthetic modification of **(1)** with NBS. Product distributions are calculated by ^1H NMR spectroscopy of digested samples.

				Solvent Mixture (ml)		Product Distribution (%)		
**Entry**	**mmol alkene in (1)**	**NBS eq.**	**DPT eq.**	**MeCN**	**H_2O**	**(1)**	**(1-Br$_2$)**	**(1-Br-OH)**
1	0.053	5	0	5	0	45	55	0
2	0.053	15	0	5	0	0	100	0
3	0.053	5	0.1	5	0	38	50	12
4	0.053	15	0.1	5	0	21	62	17
5	0.053	5	0	3	2	29	29	42
6	0.053	5	0.1	3	2	0	51	49
7	0.053	5	0.1	3	3	0	33	67
8	0.053	5	0.1	3	4	0	35	65
9	0.053	3	0.1	3	3	0	34	66
10	0.053	2	0.1	3	3	0	37	63
11	0.264	5	0.1	15	10	0	43	57

Addition of 15 equivalents of NBS (Entry 2) was necessary to quantitatively brominate **(1)**. Addition of *N,N'*-diphenylthiourea (DPT), which is known to promote bromohydrination, resulted in small amounts of the bromohydrinated product **(1-Br-OH)** when no water was added to the reaction (Entry 3), presumably resulting from excipient water. Increased amounts of NBS (Entry 4) gave slightly better conversion but still a low yield of **(1-Br-OH)**. Addition of water (Entry 5) gave significantly more bromohydrinated product, while the presence of water and DPT resulted in full conversion and up to 67% bromohydrination (Entries 6-8). Similar yields of **(1-Br$_2$)** and **(1-Br-OH)** could be achieved with as little as two equivalents of NBS in the reaction (Entries 9-10), and we found the reaction could also be scaled up with a similar product distribution (Entry 11). In our attempts to selectively bromohydrinate **(1)**, the judicious adjustment of reaction conditions to facilitate the isolation of **(1-Br-OH)** did facilitate quantitative conversion of **(1)**, but we were unable to exclude the formation of **(1-Br$_2$)**.

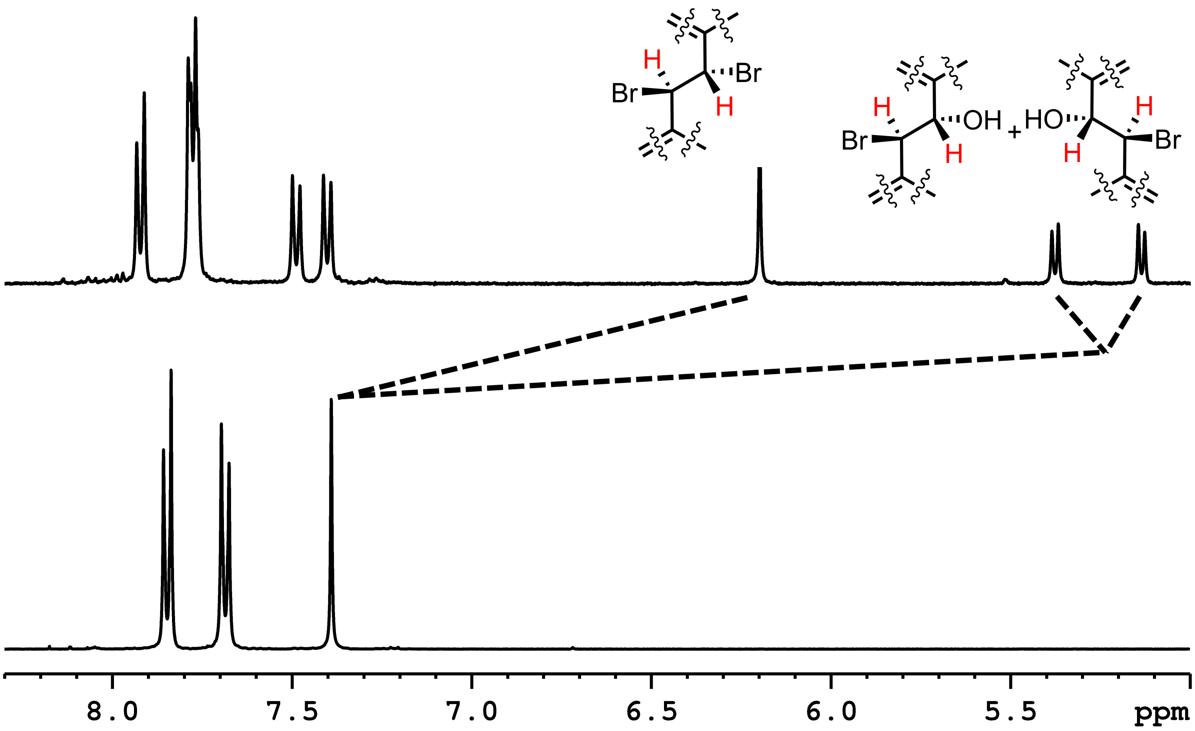

Figure S1. Partial ^{1}H NMR spectra (DMSO-d_6/D_2SO_4) of acid digested samples of **(1)** (bottom) and the material after reaction with NBS/DPT (Table S1, Entry 11) (top). The stacked spectra clearly show that **(1)** has been completely converted into both **(1-Br$_2$)** and **(1-Br-OH)**.

Figure S1 shows a typical ^{1}H NMR spectrum of a bromohydrination attempt (Entry 11 from Table S1). The aromatic protons are overlaid for the two products, **(1-Br$_2$)** and **(1-Br-OH)**, in the top ^{1}H NMR spectrum. The presence of the singlet at around 6.2 ppm is characteristic of bromoalkane protons, while the presence of two doublets at around 5.2-5.3 ppm reflects the loss of symmetry in the enantiomeric *(R,S/S,R)* bromohydrinated products **(1-Br-OH)**. No succinimidyl by-products are observed in the ^{1}H NMR spectra of the digested MOFs – presumably they are removed by the washing procedure.

Although useful for providing information regarding the conversion, ^{1}H NMR spectroscopic data were unable to provide any information on the retention of crystallinity of the MOF, although a solid phase reaction is suggested by the stereoselectivity that is observed. However, this does not definitively prove that the reaction is occurring in a postsynthetic manner. Therefore, a typical bromohydrination reaction was scaled up (Entry 11) to allow the product to be analysed by powder X-ray diffraction (PXRD) (Figure S2).

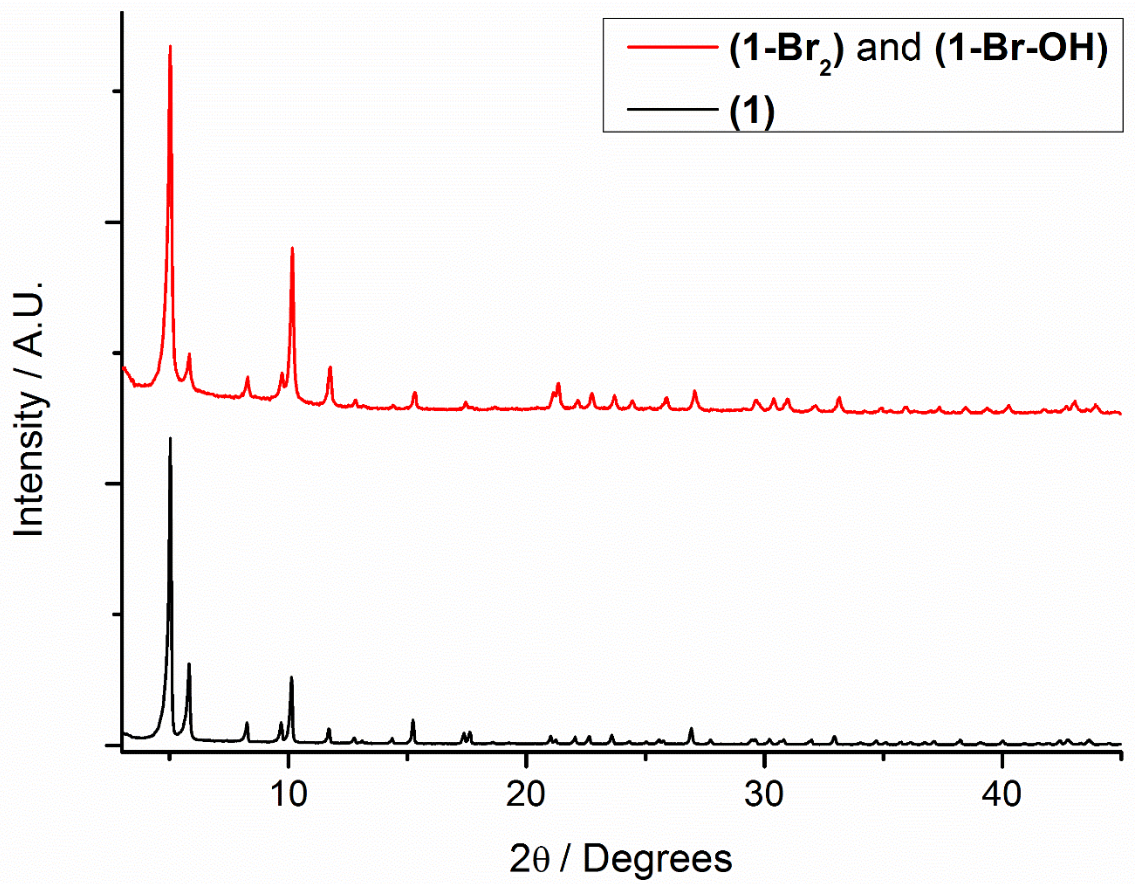

Figure S2. Comparison of the PXRD pattern of **(1)** with that of its postsynthetically derived products obtained using NBS.

Comparison of the PXRD patterns of **(1)** and its postsynthetically bromohydrinated/brominated products highlights the high mechanical stability of the MOF, as it is able to withstand a change in geometry of integral ligand atoms, as the central carbon atoms hybridisation changes from sp^2 to sp^3. The chemical stability of **(1)** is also apparent, as the MOF remains crystalline in the presence of water, which is well known to result in the collapse of many late transition metal MOFs.

Whilst NBS has allowed us to bromohydrinate **(1)**, we have chosen Br_2 as a favoured brominating agent for Zr-MOFs bearing integral unsaturated functionalities.

.

S5. Bulk Postsynthetic Bromination of (3)

Upon consideration of the attempts to postsynthetically brominate **(1)** using either NBS or neat bromine, it is apparent that the greatest success has been obtained using bromine as the brominating agent. Therefore, neat bromine was selected for subsequent brominations of **(3)**.

Bulk Bromination of **(3)**

Bulk powder of **(3)** (0.100 g, 0.50 mmol triple bond, 1 eq) was added to a 50 ml reagent bottle and left to stand in 15 ml $CHCl_3$ overnight. The $CHCl_3$ was removed and fresh $CHCl_3$ added, followed by the addition of bromine (385 μl, 7.52 mmol, 15 eq). The bottle was sealed before being stored in the dark for a period of 48 hours. The reaction product **(3-Br$_4$)** ($[Zr_6O_4(OH)_4(bdb\text{-}Br_4)_6]_n$) was collected by centrifugation and washed multiple times with fresh $CHCl_3$, before being dried under vacuum. Bromine analysis: 44.3% calculated; 43.1% found.

Bromination of **(3)** to **(3-Br$_4$)** was followed by liquid NMR spectroscopy using DMSO-*d$_6$*/D_2SO_4 mixtures for digestion of the MOF (Figures S3 and S4). The presence of D_2SO_4 alters the pH of the NMR solution which in turn affects the chemical shifts of the signals from sample to sample, meaning that they cannot be compared directly between samples, but it is however still possible to gain an understanding of the species that are present in solution.

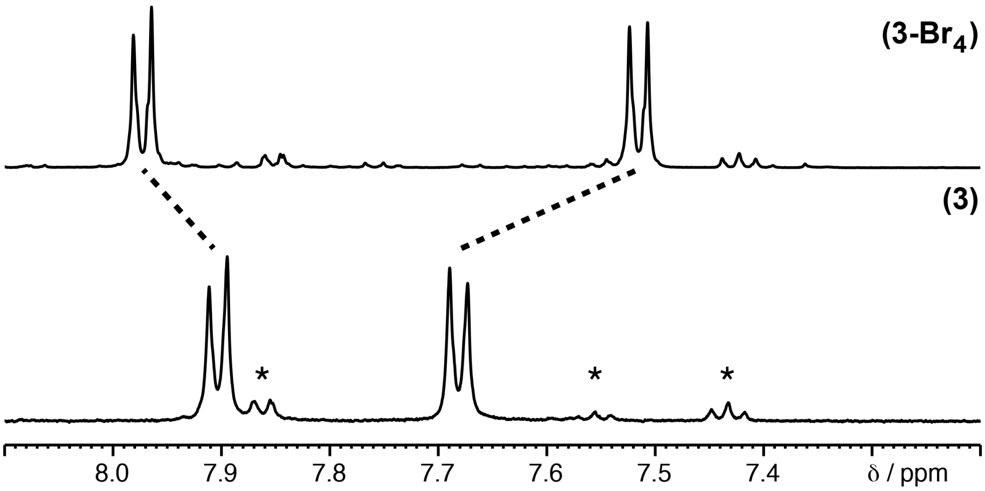

Figure S3. Stacked ^{1}H NMR spectra (DMSO-*d$_6$*/D_2SO_4) of acid digested samples of **(3)** vs **(3-Br$_4$)**. Peaks marked with an asterix represent protons corresponding to benzoic acid, which was used as a modulator during synthesis of **(3)**.

The ^{1}H NMR spectrum of **(3)** (Figure S3) contains small quantities of benzoic acid, which is also carried through to the brominated product **(3-Br$_4$)** suggesting that it is located at defect sites or becomes trapped in the pores of **(3)** during synthesis. There is limited information available from the ^{1}H NMR spectroscopic data due to the presence of only aromatic protons with the same splitting patterns in both the parent and brominated MOFs, although the increased splitting of the peaks that is observed is indicative of quantitative bromination to a single species, which we assume to be *trans,trans*-bdb-Br$_4$-H$_2$ due to the steric restrictions imposed on the linker within the MOF. ^{13}C NMR spectroscopy (Figure S4) was used to provide greater insight into the product distribution obtained.

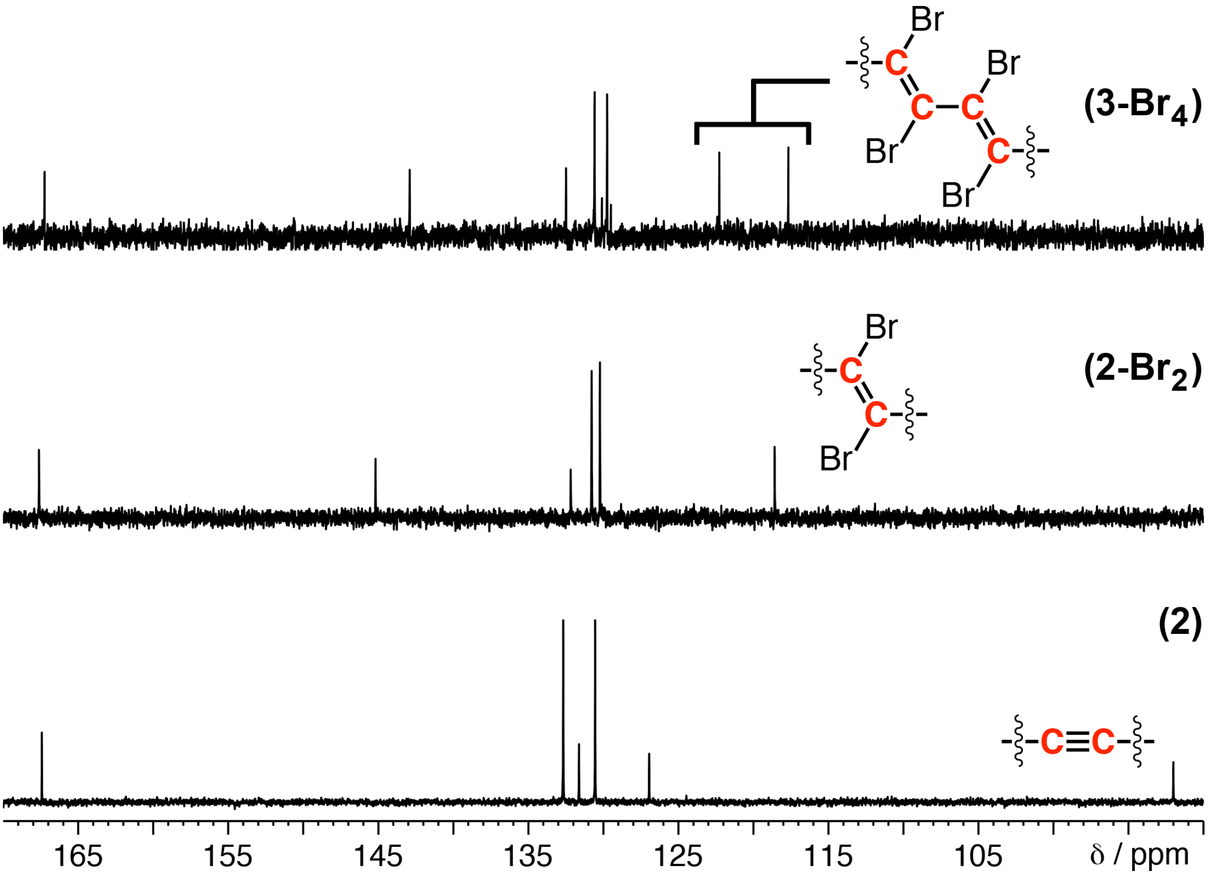

Figure S4. Stacked ^{13}C NMR spectra (DMSO-d_6/D_2SO_4) comparing acid digested samples of **(2)**, **(2-Br$_2$)** and **(3-Br$_4$)**.

Previously, ^{13}C NMR spectroscopy provided definitive evidence of the stereoselective bromination of **(2)** to **(2-Br$_2$)** due to the occurrence of only six peaks for both species, confirming that only one of the two possible isomers had been obtained.[S8] Comparison with liquid phase bromination of the ligand contained within **(2)**, as well as single crystal X-ray

crystallography, determined that *trans*-edb-Br_2-H_2 was formed exclusively. Upon bromination, the dramatic upfield shift of the alkyne carbon from δ = 92.0 ppm to δ = 118.2 ppm provided conclusive evidence for the formation of the *trans*-dibromoalkene product, owing to the geometrical constraints of the MOF.

Likewise, in the ^{13}C NMR spectrum of **(3-Br$_4$)** (Figure S4) we observe seven signals which correspond to the brominated product, alongside two small peaks around δ = 130 ppm which represent aromatic C-H signals of residual benzoic acid. The very close correlation of the chemical shifts for the *trans*-dibromoalkene carbon atoms between **(2-Br$_2$)** and **(3-Br$_4$)** (δ = 118.2 ppm vs δ = 117.6 ppm) suggests that the formation of the single species is the *trans,trans*-bdb-Br_4-H_2 product – that is that the bromination of **(3)** occurs stereoselectively. Direct comparison with the ^{13}C NMR spectrum of **(3)** was not possible, due to its very poor solubility.

In addition to the analytical techniques described in the manuscript, thermogravimetric analysis (TGA) was used to monitor the effect of bromination on the thermal stability of **(3)**. The TGA profile, collected under air, of **(3)** evidences its high thermal stability, with a major mass loss observed at around 460 °C, after losing ~5-10% mass which can be assigned to the exclusion of solvent molecules (Figure S5, overleaf). Upon comparison of the brominated material **(3-Br$_4$)** with the parent material, it is evident that a two-step profile is observed (excluding solvent loss), with the first step representing debromination. Upon comparison of the profiles of **(3)** and **(3-Br$_4$)** it is clear that the brominated material is stable at higher temperatures, with the second pronounced mass loss now observed above 500 °C.

Assuming that the material prior to debromination represents the fully evacuated MOF, then the percentage mass loss during the first step can be used to provide an initial understanding of the bromine content of the MOFs. The mass loss during the first step of **(3-Br$_4$)**, between ~200-450 °C, corresponds to a mass loss of ~26.4%, whilst the bromine content can be calculated to be 44.3%. This observation is inconsistent with the very close correlation found from elemental bromine analysis (44.3% calculated; 43.1% found) and the spectroscopic evidence which suggest quantitative bromination. Therefore we propose that in the case of **(3-Br$_4$)** it is not possible to separate the two mass losses for debromination and framework collapse using thermal analysis, hence there is a temperature range in which both processes are occurring simultaneously.

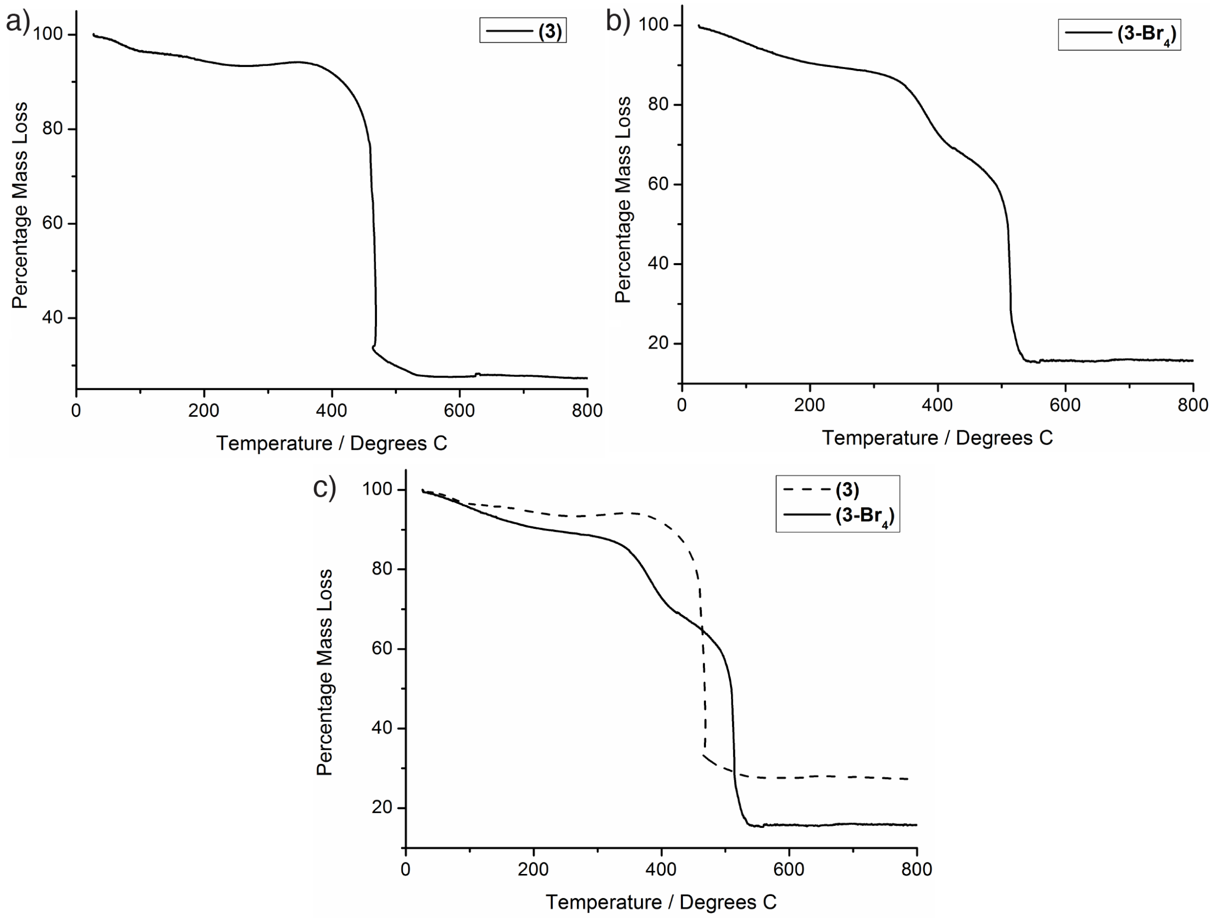

Figure S5. Thermogravimetric analysis profiles of a) **(3)**, and b) **(3-Br$_4$)** obtained under an air atmosphere. c) An overlaid comparison of the two illustrates the effect of bromination on thermal stability.

This hypothesis is reinforced by the total mass loss observed between 200-550 °C of ~82.9%, which matches exactly the theoretical mass loss expected upon decomposition of **(3-Br$_4$)** to zirconium dioxide (Table S2). The mass loss in this temperature range for **(3)** is also very close to that expected for complete conversion to ZrO_2.

Table S2. Summary of the expected (assuming complete conversion to ZrO_2) and observed mass losses obtained from TGA measurements for the parent **(3)** and the brominated product **(3-Br$_4$)**.

MOF	Formula	Expected mass loss %	Observed mass loss %
**(3)**	$C_{108}H_{52}O_{32}Zr_6$	69.3	70.7
**(3-Br$_4$)**	$C_{108}H_{52}Br_{24}O_{32}Zr_6$	82.9	82.9

S6. Solution Phase Bromination of bdb-Me_2

Solution phase bromination of bdb-Me_2, the dimethyl ester of the linker bound within **(3)**, was performed to provide a comparison of the product distribution, in terms of stereoselectivity, obtained when the brominations are carried out on the solid phase MOFs. The bromination was carried out on the diester of the MOF linker as it exhibits much greater solubility in chloroform than the dicarboxylic acid itself.

Synthesis of bdb-Br_4-Me_2

The diester bdb-Me_2 (0.060 g, 0.19 mmol, 1 eq) was added to a 50 ml reagent bottle and dissolved in 10 ml $CHCl_3$ by stirring. Bromine (292 µl, 5.70 mmol, 30 eq) was added and the jar was sealed and placed in the dark whilst stirring for 84 hours. After this time, the reaction mixture was diluted and quenched by washing with 1 M aqueous sodium thiosulfate (3 x 20 ml). The resultant yellow solution was dried over $MgSO_4$, filtered and the solvent removed under reduced pressure to yield a light coloured oil (0.121 g, 0.19 mmol, 100%). HRMS (ESI) calculated for $C_{20}H_{14}Br_4NaO_4$ $(M+Na)^+$ 656.7523, found *m/z* 656.7518.

The mass spectrometry results indicate that the tetrabromodialkene species has been synthesised, with no evidence of any partially brominated products. This knowledge helped with the assignment of the ^{1}H NMR spectrum (Figure S6, overleaf), which indicates the presence of more than one compound. The presence of three sets of resonances combined with the disappearance of the peaks relating to bdb-Me_2 provides evidence that the bromination has occurred quantitatively and that the product contains a mixture of three geometric isomers. ^{13}C NMR spectroscopic data was collected for the product, with a large number of resonances again suggesting the presence of multiple geometric isomers, although they could not be confidently assigned due to the similarity of their chemical shifts.

The ^{1}H NMR spectrum of bdb-Br_4-Me_2 indicates the presence of three species which we have assigned to be the *trans,trans*, *cis,trans* and *cis,cis* geometric isomers. Using integral ratios, we can determine that the relative product distribution is 50:40:10 (*trans,trans*:*cis,trans*:*cis,cis*). We can confidently assign the resonances associated with the *trans*,*cis* isomer, as it exhibits lower symmetry and therefore twice the number of signals as the *trans*,*trans* and *cis*,*cis* isomers. Based on the expected steric preference for the formation

of the *trans*,*trans* over the *cis*,*cis* isomer, we have assumed the more prevalent of the two symmetrical species is the *trans,trans* isomer. In any case, it is clear that the liquid phase bromination occurs non-stereoselectively.

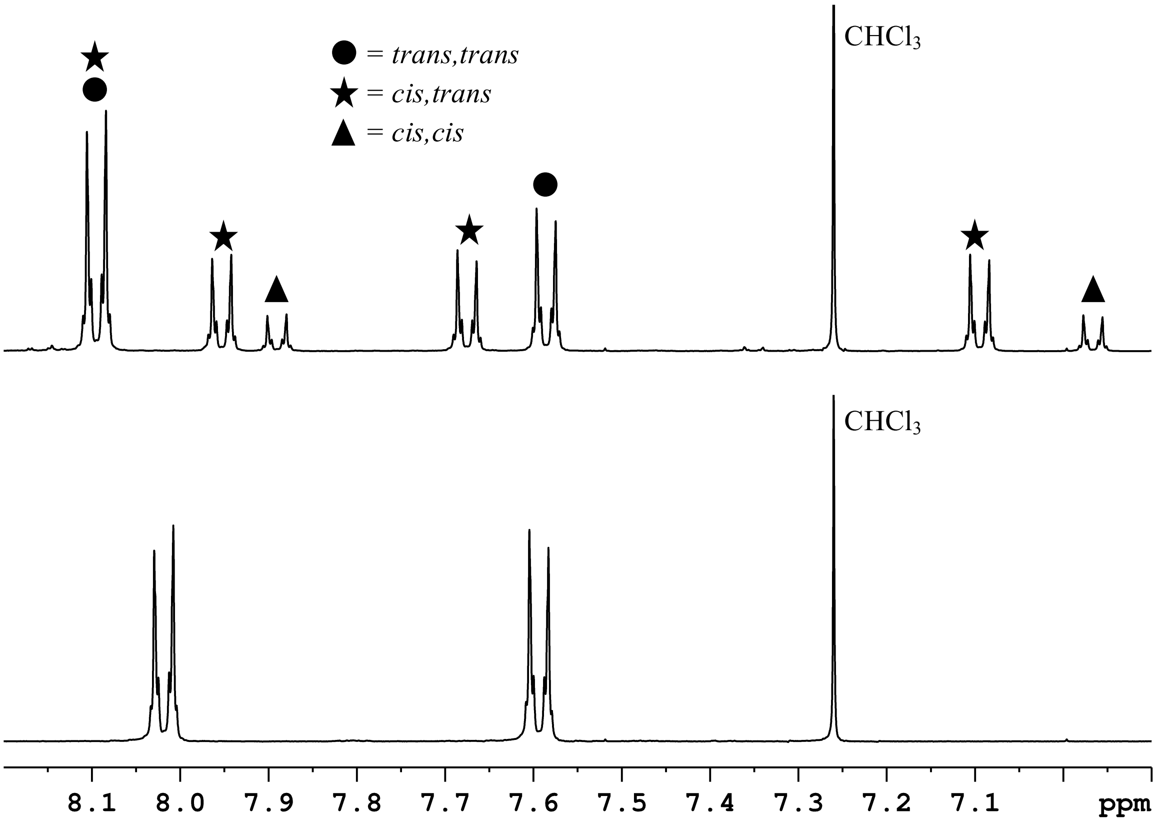

Figure S6. Stacked partial ^{1}H NMR spectra ($CDCl_3$) of bdb-Me_2 and its brominated products, the isomers of bdb-Br_4-Me_2.

S7. Bulk Postsynthetic Bromination of (4) and (5)

As with **(3)**, neat bromine was chosen to brominate the interpenetrated MOFs **(4)** and **(5)**, as it was the most efficient brominating agent.

Bulk Bromination of **(4)**

Bulk powder of **(4)** (0.150 g, 0.63 mmol triple bond, 1 eq) was added to a 50 ml reagent bottle and immersed in 25 ml $CHCl_3$. Bromine (965 μl, 18.84 mmol, 30 eq) was added and the bottle was sealed before being stored in the dark for a period of 72 hours. The reaction product **(4-Br$_4$)** $[Zr_6O_4(OH)_4(peb\text{-}Br_4)_6]_n$ was collected by centrifugation and washed multiple times with fresh $CHCl_3$, before being dried under vacuum. Bromine analysis: 40.1% calculated; 41.9% found.

Bulk Bromination of **(5)**

Bulk powder of **(5)** (0.065 g, 0.23 mmol triple bond, 1 eq) was added to a 50 ml reagent bottle and immersed in 15 ml $CHCl_3$. Bromine (355 μl, 6.93 mmol, 30 eq) was added and the bottle was sealed before being stored in the dark for a period of 72 hours. The reaction product **(5-Br$_4$)** $[Hf_6O_4(OH)_4(peb\text{-}Br_4)_6]_n$ was collected by centrifugation and washed multiple times with fresh $CHCl_3$, before being dried under vacuum. Bromine analysis: 36.1% calculated; 33.3% found.

Comparison of the PXRD patterns of **(4)** and **(5)** with their brominated products **(4-Br$_4$)** and **(5-Br$_4$)** reveals that crystallinity is retained during bromination, with noticeable differences observed between the PXRD patterns of the starting materials and the halogenated products (Figure S7, overleaf). Upon bromination of the alkynes to dibromoalkene units, there is an associated mechanical contraction, causing the peaks of the daughter materials to shift to slightly higher values of 2θ relative to their parents. The relative intensities and shapes of the peaks are also observed to change upon bromination, which together suggests that quantitative conversion has been achieved. Very similar patterns are observed for the brominated products **(4-Br$_4$)** and **(5-Br$_4$)**, suggesting that they are structurally similar and that bromination occurs in a facile manner regardless of the metal used in the synthesis of the MOF.

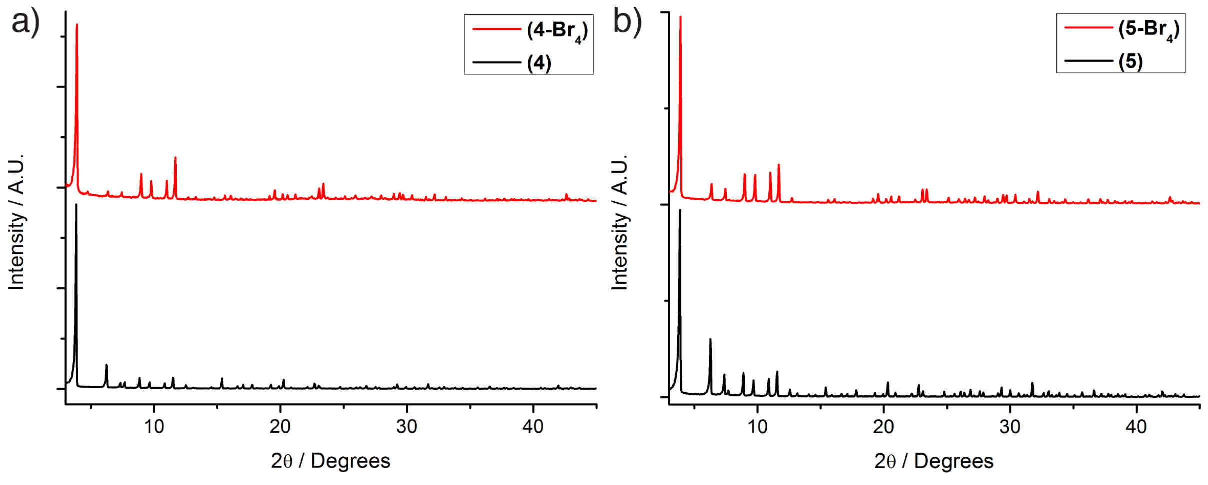

Figure S7. Stacked PXRD patterns highlighting the differences upon bromination between a) **(4)** and **(4-Br$_4$)**, and b) **(5)** and **(5-Br$_4$)**.

^{1}H NMR spectroscopy was again used to monitor the extent of bromination of the MOFs. In our experience the addition of bromine to the unsaturated carbon atoms of the MOF ligands usually increases linker solubility in the DMSO-d_6/D_2SO_4 solvent mixtures used for NMR spectroscopy. The converse is true between peb-H_2 and peb-Br_4-H_2 (the ligands contained within **(4)** and **(5)** and their brominated products **(4-Br$_4$)** and **(5-Br$_4$)**) with ligand solubility decreasing dramatically upon bromination. The limited solubility of peb-Br_4-H_2 made routine NMR spectroscopic analysis very difficult, and so small amounts of triethylamine were added to the solutions to solubilise the ligands. As a result of the low solubility it was not possible to follow the bromination of **(4)** and **(5)** using ^{13}C NMR spectroscopy, with only limited information available from ^{1}H NMR spectroscopic data (Figures S8 and S9, overleaf).

The comparisons of both **(4)** with **(4-Br$_4$)** and **(5)** with **(5-Br$_4$)** show the formation of only a single species upon exposure to bromine, with changes in the chemical shifts observed. Both comparisons suggest the formation of a single brominated species of the same symmetry as the parent material, complementing the other analytical techniques utilised in the study.

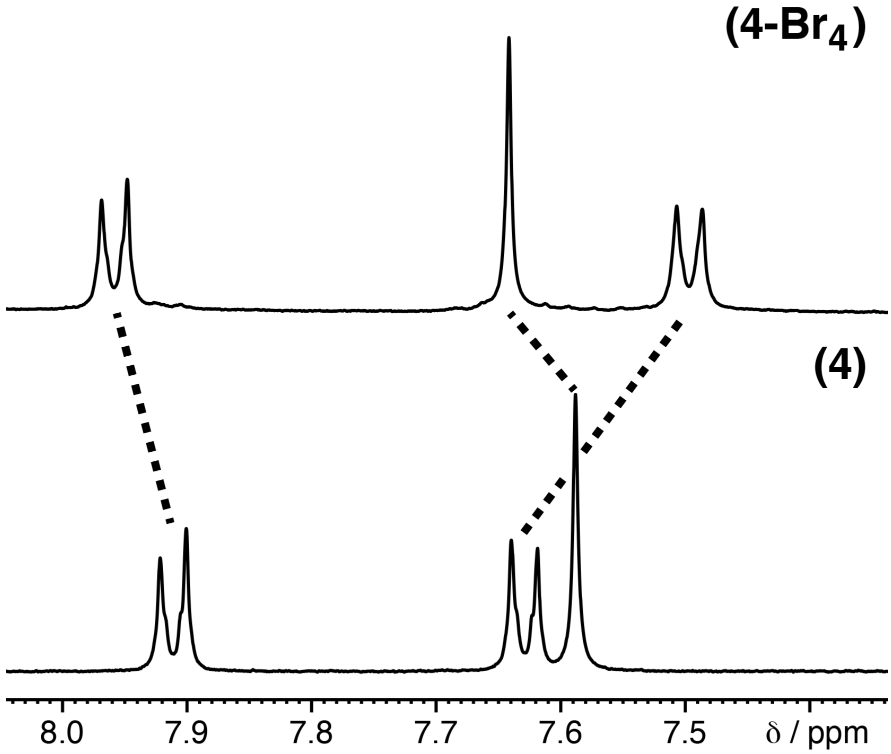

Figure S8. Stacked ^{1}H NMR spectra (DMSO-d_6/D_2SO_4) of acid digested samples of **(4)** vs **(4-Br$_4$)**.

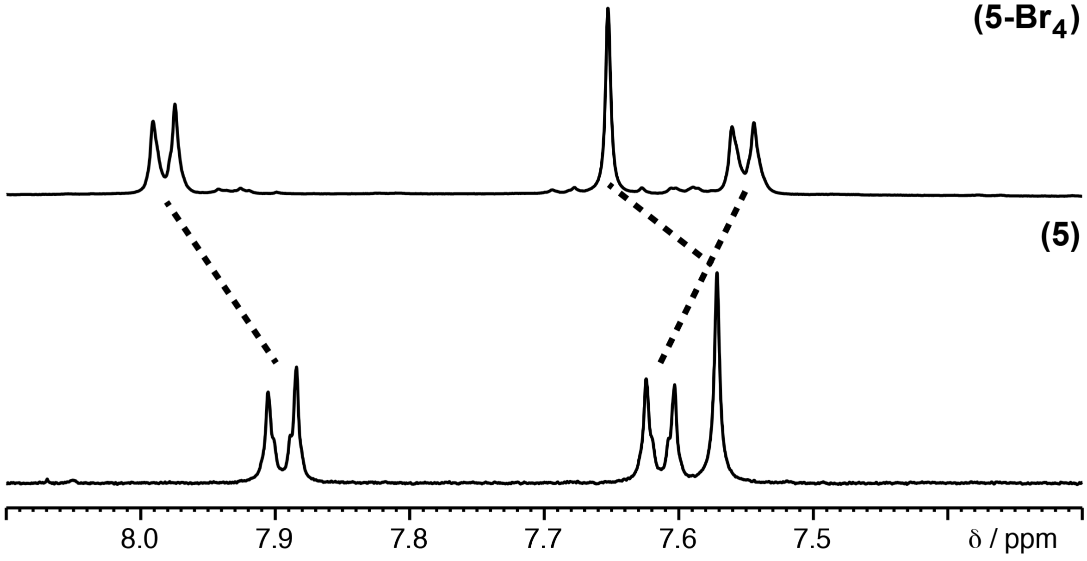

Figure S9. Stacked ^{1}H NMR spectra (DMSO-d_6/D_2SO_4) of acid digested samples of **(5)** vs **(5-Br$_4$)**.

Raman spectra of the MOFs are also indicative of quantitative bromination (Figure S10, overleaf). In the parent MOFs, the signals at ~2220 cm^{-1} for **(4)** and **(5)** are characteristic of alkyne units. During bromination, these functional units are converted to dibromoalkene moieties, therefore it is expected that these peaks corresponding to C≡C bonds should no

longer be visible if the transformation has occurred quantitatively. In both brominated samples this is the case, with broadening of the alkene peak at ~1600 cm^{-1} observed, suggesting the appearance of a new alkene moiety, although the rapid damaging of the brominated samples by the Raman laser make this difficult to distinguish, particularly for **(5-Br$_4$)**. It is clear however, that the signal associated with the alkyne unit is no longer present, indicating full conversion.

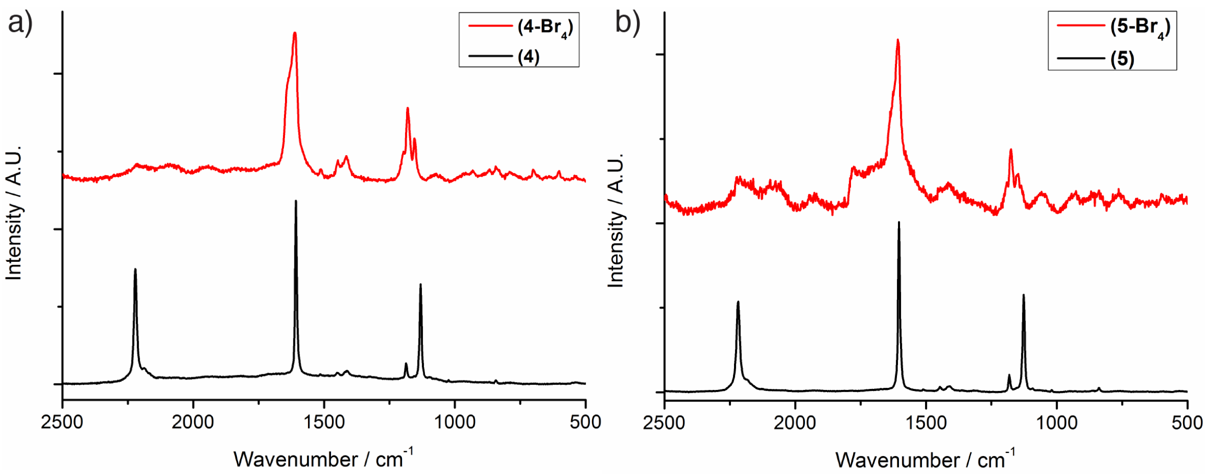

Figure S10. Stacked Raman spectra highlighting the differences between a) **(4)** and its brominated product **(4-Br$_4$)** and b) **(5)** and its brominated product **(5-Br$_4$)**. The Raman laser rapidly damaged the brominated materials, hence the poor quality of their spectra.

Thermogravimetric analysis in air (Figure S11, overleaf) was again employed to provide an understanding of the thermal stability of the interpenetrated MOFs and their brominated products. Both **(4)** and **(5)** demonstrate high thermal stabilities, with major mass losses observed at around 470 °C, as is expected for zirconium and hafnium MOFs.

As expected, both **(4-Br$_4$)** and **(5-Br$_4$)** demonstrate two-step profiles, with the first mass loss observed between 200-465 °C in **(4-Br$_4$)** representing a mass loss of ~39.1%, in very close agreement with the theoretical bromine content of 40.1%. Likewise, the first step in **(5-Br$_4$)** between 200-475 °C represents a mass loss of ~34.9%, in close agreement with the theoretical Br content of 36.1% The thermal stabilities of **(4)** and **(5)** following debromination correlates well with the observed thermal profiles of the parent materials.

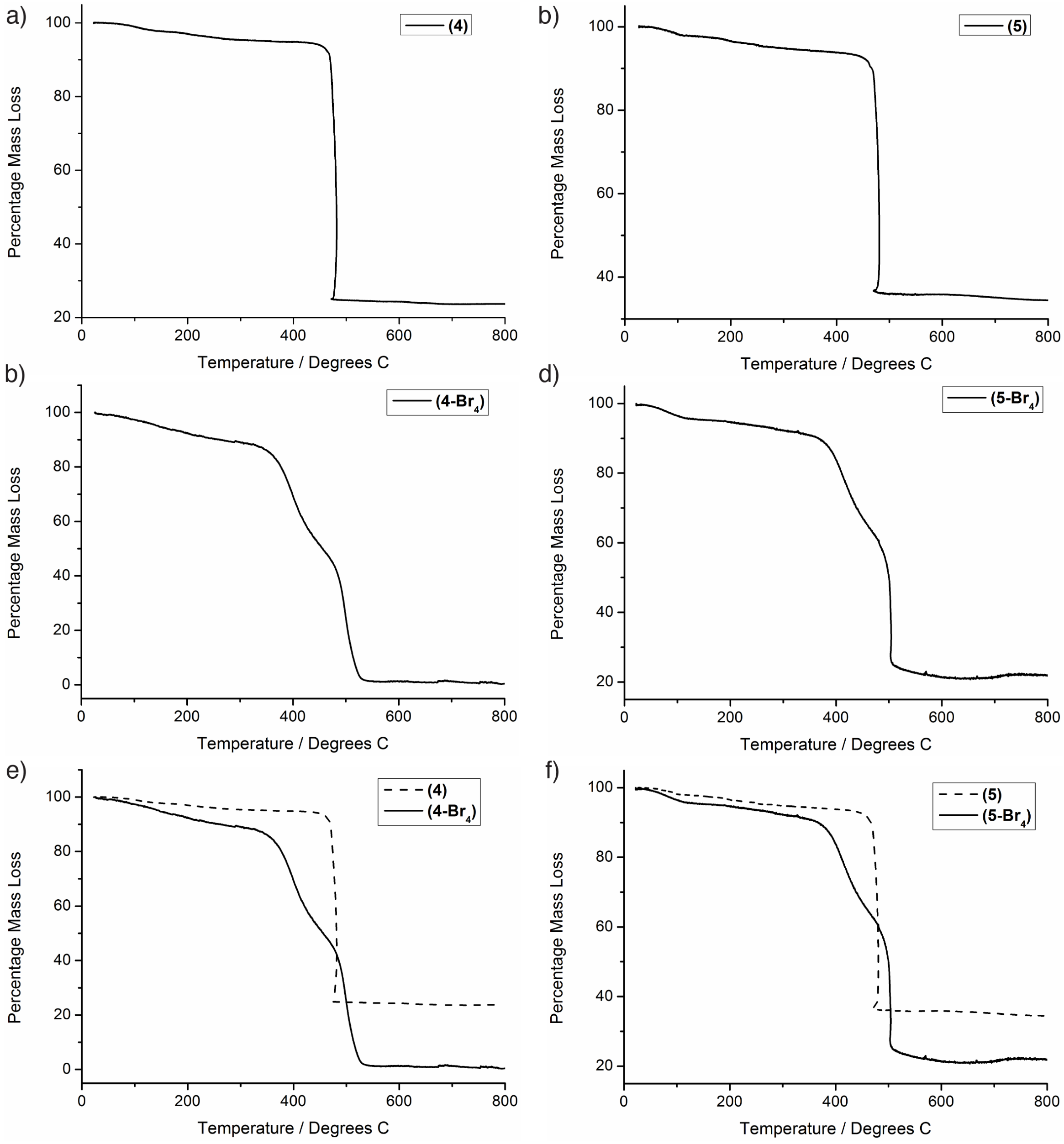

Figure S11. Thermogravimetric analysis profiles of a) **(4)**, b) **(5)**, c) **(4-Br$_4$)** and d) **(5-Br$_4$)** obtained under an air atmosphere. Overlaid comparisons for the conversion of e) **(4)** to **(4-Br$_4$)** and f) **(5)** to **(5-Br$_4$)** illustrate the effect of bromination.

Excellent correlations are observed between the total experimental mass losses and the expected mass losses should the starting MOFs and brominated materials be completely converted to the corresponding metal oxides (Table S3, overleaf). This further indicates that bromination has occurred quantitatively.

Table S3. Summary of the expected (assuming complete conversion to ZrO_2/HfO_2) and observed mass losses obtained from TGA measurements for the parent MOFs **(4)** and **(5)** as well as their brominated products **(4-Br$_4$)** and **(5-Br$_4$)**.

MOF	Formula	Expected mass loss %	Observed mass loss %
**(4)**	$C_{144}H_{76}O_{32}Zr_6$	74.2	74.5
**(4-Br$_4$)**	$C_{144}H_{76}Br_{24}O_{32}Zr_6$	84.5	85.0
**(5)**	$C_{144}H_{76}Hf_6O_{32}$	62.7	62.6
**(5-Br$_4$)**	$C_{144}H_{76}Br_{24}Hf_6O_{32}$	76.2	76.1

N_2 adsorption isotherms were performed at 77 K (Figure S12) to gain an understanding of the porosities of **(4)** and **(5)** as well as their brominated products, whilst also examining the effect of interpenetration.

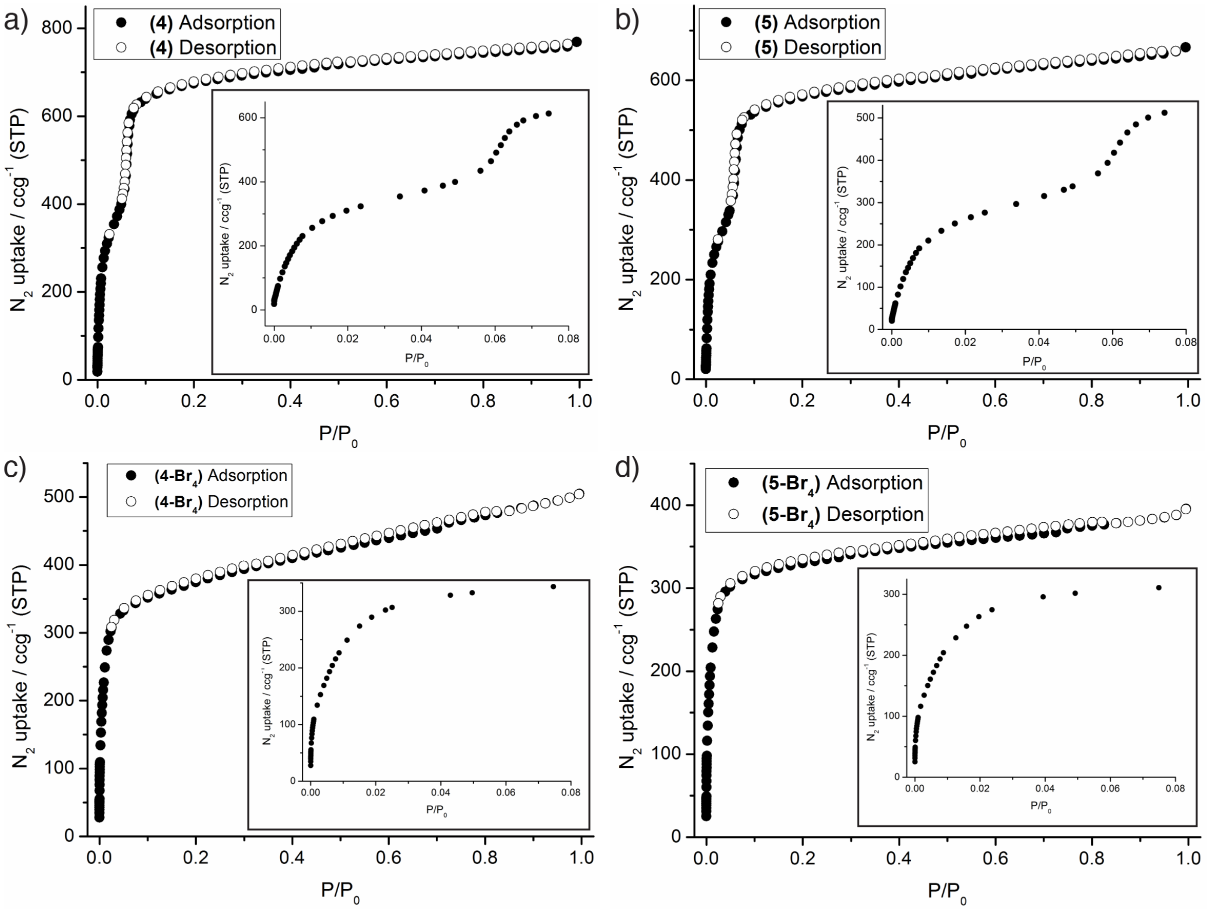

Figure S12. N_2 adsorption isotherms at 77 K of a) **(4)**, b) **(5)**, c) **(4-Br$_4$)** and d) **(5-Br$_4$)**. The insets are expanded illustrations of the low pressure region adsorption data.

The BET surface area of **(4)** is measured to be 2650 m^2g^{-1}, which is lower than **(3)** as a direct result of framework interpenetration reducing the void free space of the MOF. The adsorption isotherms of **(4)** and **(5)** are very similar in shape, which is unsurprising considering the MOFs are isostructural. The BET surface area of **(5)** is 2250 m^2g^{-1}, which is in good agreement with the surface area of **(4)**, taking into account the increased mass of the material and thus lower gravimetric surface area as a result of Hf replacing Zr.

The adsorption isotherms of **(4-Br$_4$)** and **(5-Br$_4$)** are completely different from their parents, with a loss of the stepwise adsorption profile. Upon bromination of **(4)** to **(4-Br$_4$)** the BET surface area decreases by 46% from 2650 m^2g^{-1} to 1440 m^2g^{-1}, however the increase in mass due to the bromine atoms cannot fully account for this reduction (theoretical decrease 40%). Similarly, the BET surface area of **(5)** decreases from 2250 m^2g^{-1} to 1320 m^2g^{-1} upon bromination to **(5-Br$_4$)**, corresponding to a reduction of 41%, whilst the theoretical decrease due to the mass of bromine is calculated as 36%. We attribute the additional reduction in surface areas to the mechanical contraction introduced during bromination and the presence of the bulky Br atoms that now occupy the pores of the MOF.

The mechanical contraction of the MOFs that occurs during bromination of integral framework sites is evident from pore size distributions (PSDs, Figure S13) calculated from the N_2 adsorption isotherms. The PSDs indicate significant mechanical contractions upon bromination of both the parent interpenetrated MOFs **(4)** and **(5)**, with the diameter of the main pore observed to decrease from 14.2 Å to 11.0 Å in each case.

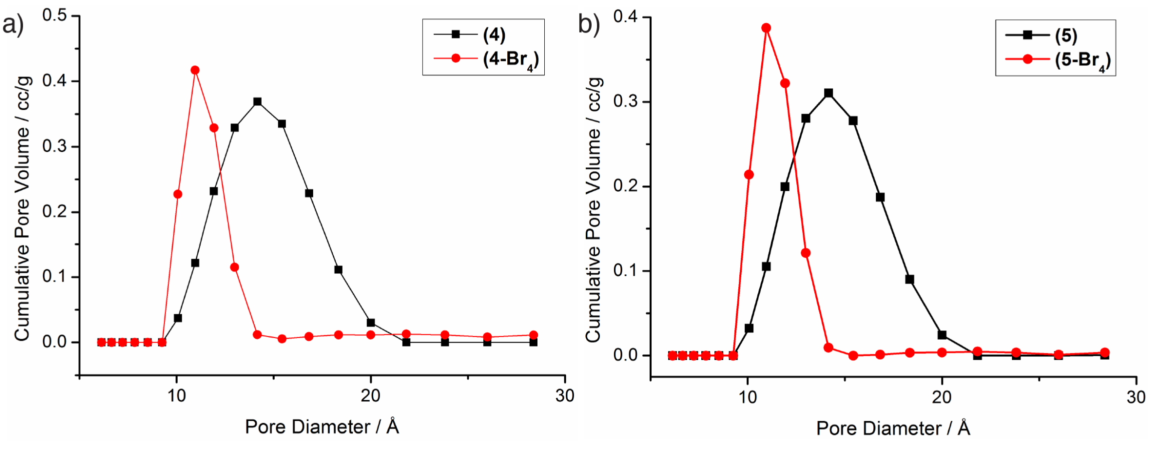

Figure S13. Pore size distribution comparisons, calculated by QSDFT, for a) **(4)** and **(4-Br$_4$)** and b) **(5)** and **(5-Br$_4$)**.

S8. Solution Phase Bromination of peb-Me_2

As with the bromination of **(3)**, the stereoselectivity of the bromination of **(4)** and **(5)** was contrasted with the solution phase bromination of the dimethyl ester, peb-Me_2, of the ligand that constitutes the interpenetrated MOFs.

Synthesis of peb-Br_4-Me_2

The diester peb-Me_2 (0.025 g, 0.06 mmol, 1 eq) was added to a 50 ml reagent bottle and dissolved in 10 ml $CHCl_3$ by stirring. Bromine (195 µl, 3.81 mmol, 64 eq) was added and the jar was sealed and placed in the dark whilst stirring for 48 hours. After this time, the reaction mixture was diluted and quenched by washing with 1 M aqueous sodium thiosulfate (3 x 20 ml). The reaction mixture was dried over $MgSO_4$, filtered and the solvent removed under reduced pressure to yield a white solid (0.043 g, 0.06 mmol, 100%). HRMS (CI) calculated for $C_{26}H_{18}Br_4O_4$ $(M)^+$ 709.7939, found *m/z* 709.7933.

Comparison of the ^1H NMR spectra of the starting material and products (Figure S14, overleaf) shows that peb-Me_2 has been quantitatively converted into peb-Br_4-Me_2 using neat bromine in conditions that resemble the reactions performed on the solid phase MOFs. The number of resonances in the ^1H NMR spectrum of peb-Br_4-Me_2 indicates the presence of more than one species, which we have again assigned as the three different geometric isomers that are possible upon formation of the dibromoalkene units. From the aromatic region of the ^1H NMR spectrum it is evident that there is one main product, but overlapping resonances make assignment tricky.

Alternatively, by inspection of diagnostic methyl ester peaks and their integrations, we can assign the products and their relative proportions in a manner similar to the previous example. As such we have found that the liquid-phase bromination results in all of the possible geometric isomers, namely *trans,trans*, *cis,trans* and *cis,cis* which are present in a 60:35:5 ratio, assuming that formation of the *trans*,*trans* isomer is preferred over the sterically more hindered *cis*,*cis* isomer. Using this information, it is also possible to assign the complex overlapping aromatic signals as corresponding to these three isomers. The limited solubility of peb-Br_4-Me_2 in routine NMR solvents prevented ^{13}C NMR spectroscopic data from being collected.

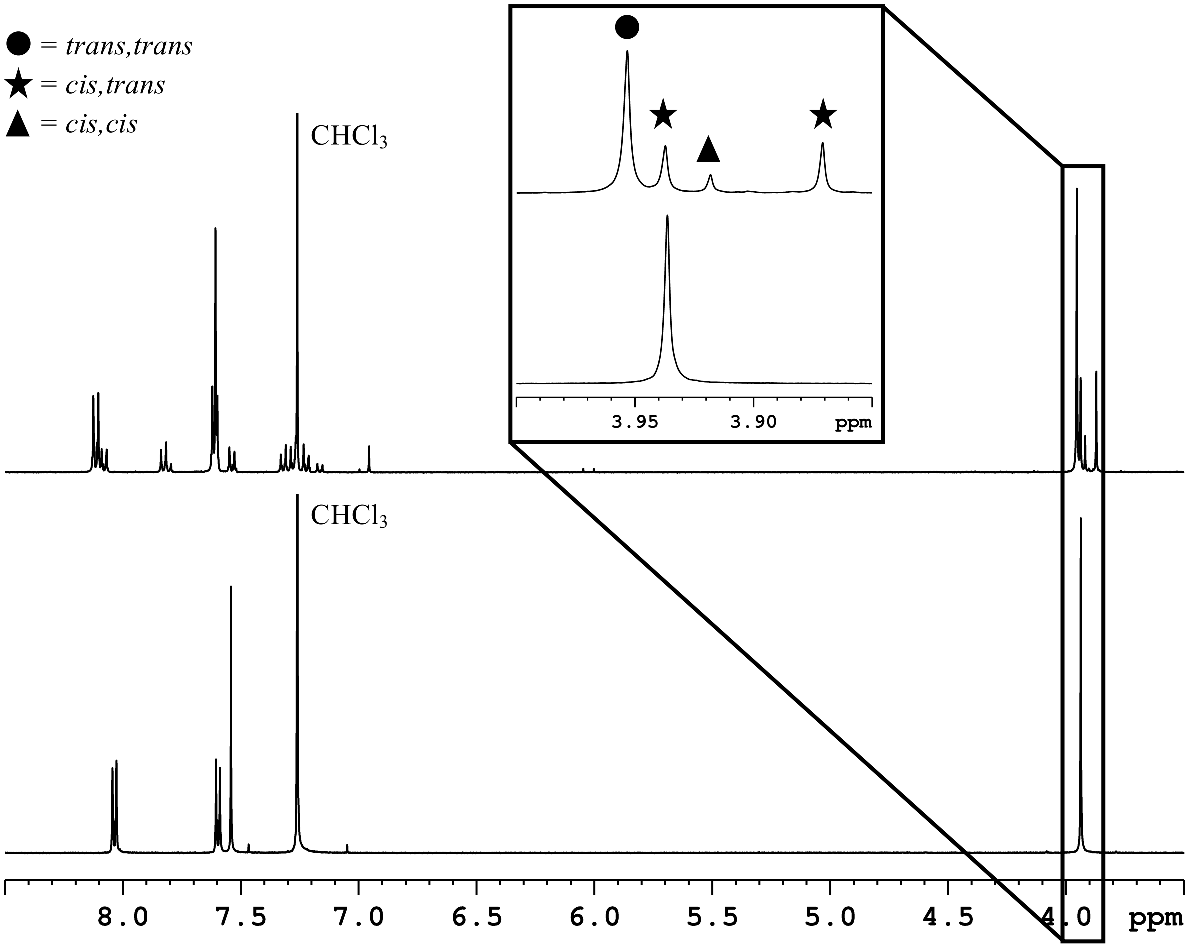

Figure S14. Stacked partial ^{1}H NMR spectra ($CDCl_3$) of peb-Me_2 and its brominated products, the isomers of peb-Br_4-Me_2.

S9. Single-Crystal to Single-Crystal Bromination of (4) and (5)

Considering our efforts towards bulk phase brominations of the new zirconium MOF **(3)** with UiO topology, and both the isoreticular interpenetrated zirconium **(4)** and hafnium **(5)** MOFs, we decided to investigate the feasibility of their transformation to **(3-Br$_4$)**, **(4-Br$_4$)** and **(5-Br$_4$)**, in turn, in a single crystal manner. The bromination of these MOFs, although it appears to be facile, does require a high degree of chemical and mechanical stability. This is due to the prolonged contact with a highly reactive bromine solution combined with the large amounts of mechanical strain that is induced during the contraction of the framework as the hybridisation of the integral carbon atoms changes from *sp* to sp^2.

Single Crystal Bromination – General Procedure

Small amounts of single crystals of the MOF still in their mother liquor were added by pipette to a 10 ml vial containing fresh DMF (3 ml). The DMF was exchanged for fresh DMF twice before being exchanged for $CHCl_3$ (3 ml). The $CHCl_3$ was exchanged for fresh $CHCl_3$ a further two times. Bromine (100 µl for **(3)**; 50 µl for **(4)** and **(5)**) was added, the vial was sealed and left to stand in the dark for 96 hours. The $CHCl_3$ was replaced multiple times with fresh $CHCl_3$. Small portions of the crystals were added to a vial containing DMF to resolvate. The DMF was exchanged for fresh DMF and the crystals left to stand.

Unfortunately, it did not prove possible to collect anything other than unit cell parameters for **(3-Br$_4$)**, likely as a result of the significant disorder and frustration imposed by the linker. The decrease in unit cell edge did, however, provide evidence of bromination and a concurrent mechanical contraction.

Single crystal diffraction data were collected and processed using CrystalClear-SM Expert 3.1 b27.[S10] The structure was solved using Superflip[S15] and refined using SHELXL[S12] within Olex2.[S13] Only the Zr atoms were refined with anisotropic atomic displacement parameters (adps), all other atoms were refined with isotropic adps and similarity restraints applied to the carbon atoms. Disorder was modelled as two 0.5 occupied sites for C7, both Br sites and C8 and C9 of the central aromatic ring (with C6 and C10 common to both orientations, see Figure S15). Distance and planarity restraints were applied. The Br atoms have large displacement ellipsoids and the residual electron density suggests that the

positions are smeared over a wider arc than has been modelled, particularly for Br2 which faces into the solvent accessible cavity. Hydrogen atoms were placed in geometrically calculated positions and included as part of a riding model, except the hydrogen atoms on C9, C10 and the OH hydrogen atoms, which were not included explicitly in the model but are included in the cell contents and all values derived from them (see Figure S15 for atom labelling).

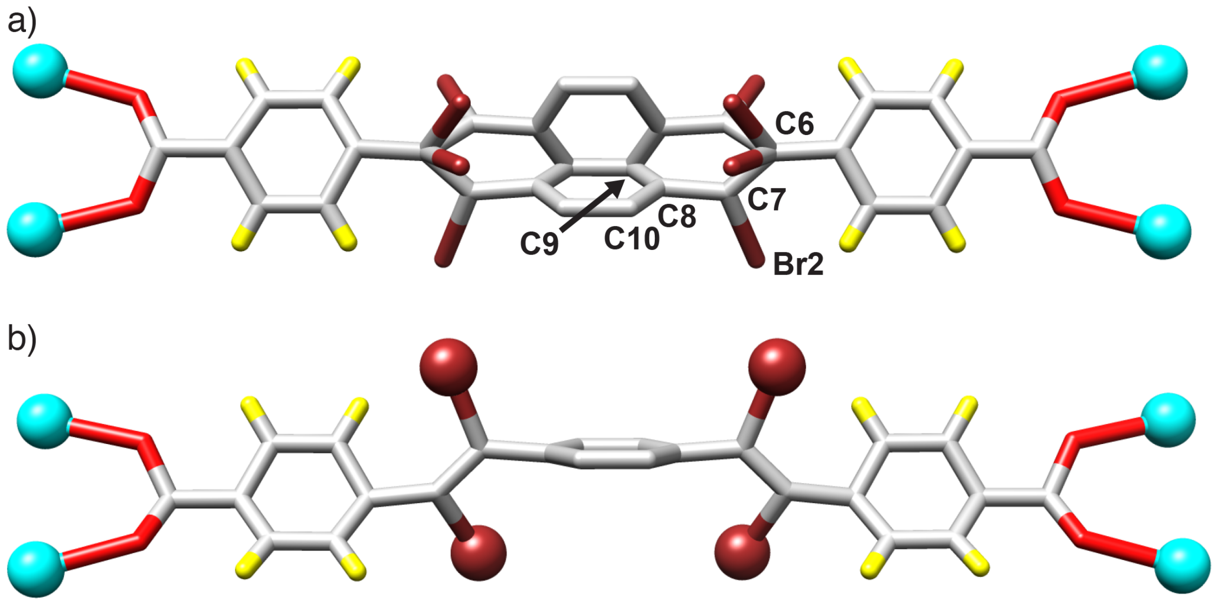

Figure S15. Representations of the peb-Br_4^{2-} linker from the crystal structure of **(4-Br_4)**, with a) disorder of the central benzene ring shown, alongside selected atom labelling, and b) with disorder removed, showing the ligand adopts a *tran*,*trans* configuration.

Approximately 44% of the cell volume is not occupied by the framework and contains diffuse and disordered solvent molecules. This electron density was accounted for using SQUEEZE within PLATON[S14] which calculated a solvent accessible volume of 26061 Å^3 containing 5123 electrons. The structure was deposited with the Cambridge Structural Database with deposition number CCDC 1443198.

Crystal data **(4-Br_4)**. $C_{144}H_{76}Br_{24}O_{32}Zr_6$, M_r = 4783.20, Cubic, a = 39.067 (7) Å, V = 59625 (32) Å^3, T = 100 K, space group Fd-3m (no. 227), Z = 8, 113804 measured reflections, 2511 unique (R_{int} = 0.095), which was used in all calculations. The final R_1 = 0.214 for 2473 observed data $R[F^2 > 2\sigma(F^2)]$ and $wR(F^2)$ = 0.565 (all data).

Single crystal diffraction data were collected using CrystalClear-SM Expert 3.1 b27[S10] and processed using CrysAlis PRO 1.171.38.41.[S11] The structure was solved using Superflip[S15] and refined using SHELXL[S12] within Olex2.[S13] Only the Zr atoms were refined with anisotropic atomic displacement parameters (adps), all other atoms were refined with isotropic adps and similarity restraints applied. Disorder was modelled as two 0.5 occupied sites for C7 and both Br sites. Distance and planarity restraints were applied. The Br atoms have large displacement ellipsoids and the residual electron density suggests that the positions are smeared over a wider arc than has been modelled, particularly for Br2 which faces into the solvent accessible cavity. Hydrogen atoms were placed in geometrically calculated positions and included as part of a riding model, except the OH hydrogen atoms, which were not included explicitly in the model but are included in the cell contents and all values derived from them. Approximately 50% of the cell volume is not occupied by the framework and contains diffuse and disordered solvent molecules. This electron density was accounted for using SQUEEZE within PLATON[S14] which calculated a solvent accessible volume of 29600 Å^3 containing 12699 electrons. The structure was deposited with the Cambridge Structural Database with deposition number CCDC 1443199.

Crystal data **(5-Br$_4$)**. $C_{144}H_{76}Br_{24}Hf_6O_{32}$, M_r = 5306.82, Cubic, a = 39.0451 (3) Å, V = 59525.0 (14) Å^3, T = 100 K, space group Fd-3m (no. 227), Z = 8, 65301 measured reflections, 3215 unique (R_{int} = 0.047), which was used in all calculations. The final R_1 = 0.122 for 4491 observed data $R[F^2 > 2\sigma(F^2)]$ and $wR(F^2)$ = 0.464 (all data).

S10. Iodination of (1), (2), (3) and (4)

Considering the reactivity of the MOFs towards bromine, we decided to investigate their potential as I_2 storage materials, which could find important applications in the nuclear industry to avoid the release of radioactive iodine.[S18] We designed two sets of experiments, firstly measuring the chemisorption of I_2 vapours by the MOFs by ^{1}H NMR spectroscopy, and secondly total iodine uptake through gravimetric experiments.

Iodine Chemisorption Experiments

In a typical reaction, **(1)**, **(2)**, **(3)** or **(4)** was added to an open-ended 1 dram vial. Separately, I_2 (15 eq per multiple bond) was added to a 30 ml screw top vial (Table S4). The small vial containing the MOF was then placed in the centre of the large vial, with the lid of the outer vial tightened to create a closed system. The experiments were allowed to stand at room temperature for the required number of days (1, 3, 7, 14 and 21 days, except for experiments containing **(4)** when data was also collected at 28 days). To stop the reaction, the small vial was removed and the contents added to a 15 ml centrifuge tube. The product was collected by centrifugation multiple times with $CHCl_3$ (10 ml) until washings were colourless, before drying under vacuum. The percentage chemisorbed I_2 was calculated from ^{1}H NMR spectroscopic integral ratios obtained from DMSO-d_6/D_2SO_4 digests of the products, as previously used to determine the extent of bromination.

Table S4. Summary of reaction quantities for I_2 chemisorption experiments.

**MOF**	**Mass (mg)**	**mmol unsaturated bond**	**mmol I_2**	**Mass I_2 (mg)**	**Equivalents I_2**
**(1)**	10	0.026	0.40	50	15
**(2)**	10	0.027	0.40	50	15
**(3)**	10	0.050	0.75	95	15
**(4)**	10	0.042	0.63	80	15

The bromination of **(1)** in chloroform is facile, with quantitative conversion achieved in the presence of as little as 5 equivalents of bromine. This is in stark contrast to the results obtained here, where iodine addition across the integral alkene bond through vapour diffusion does not occur even when exposed to 15 equivalents of iodine. This lack of reactivity is clear from the ^{1}H NMR spectroscopic data (Figure S16), with no observed change in the deterministic alkene protons observed.

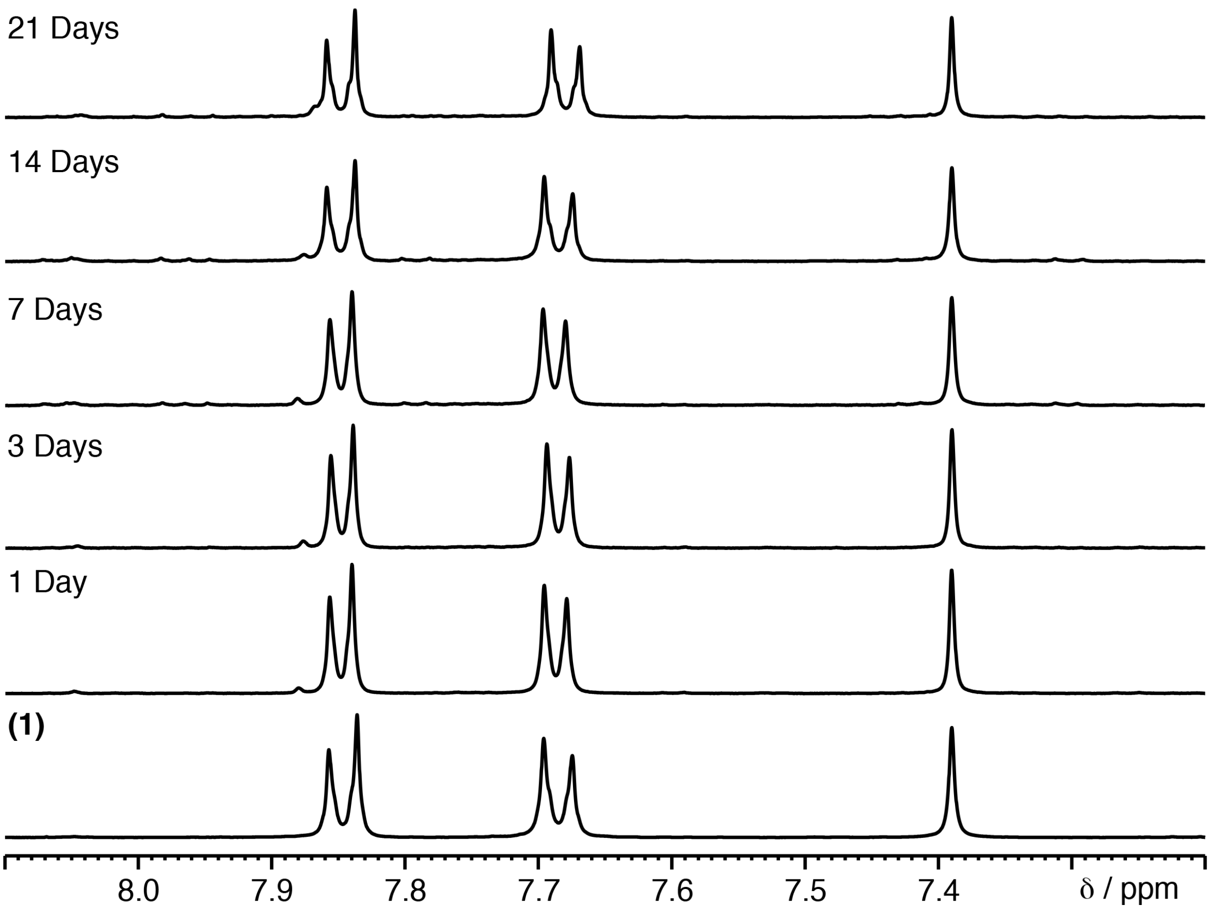

Figure S16. Stacked partial ^{1}H NMR spectra (DMSO-d_6/D_2SO_4) of **(1)** compared with samples of **(1)** exposed to I_2 vapours for a set numbers of days. The spectra were referenced by setting the alkene proton singlet to 7.39 ppm, to counteract slight signal shifts due to differences in pH resulting from acid digestions.

In contrast, ^{1}H NMR spectroscopic data reveal (Figure S17) the successful addition of iodine from the vapour phase across the alkyne bonds of the edb^{2-} ligands contained within **(2)**, building on preliminary studies which investigated iodine chemisorption at 7 days.[S8] The comprehensive study conducted here however reveals that the amount of I_2 chemisorbed continues to increase until 14 days to a maximum value of ~84% conversion of the ligand to *trans*-edb-I_2-H_2, corresponding to 57% *w/w* irreversible chemisorption of iodine.

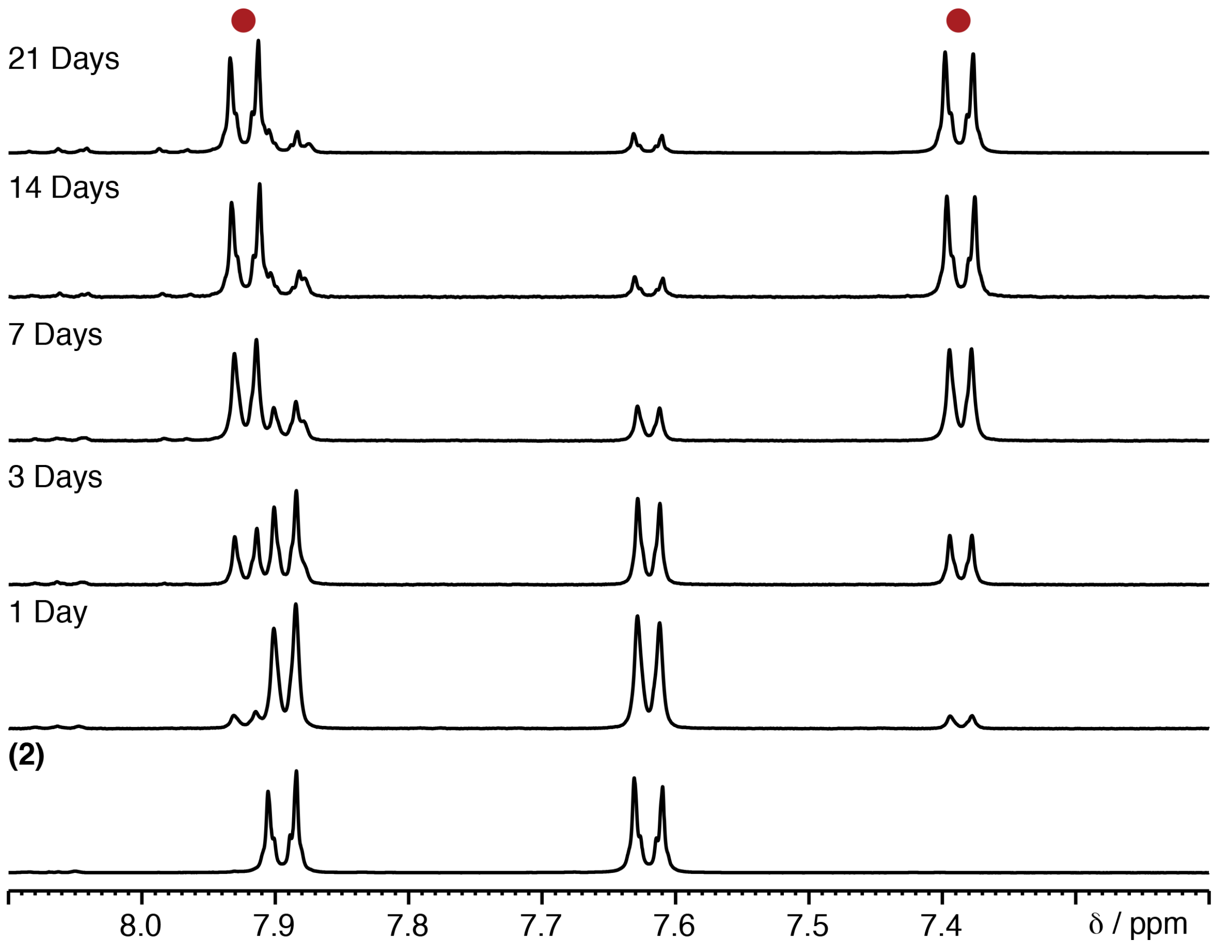

Figure S17. Stacked partial ^{1}H NMR spectra (DMSO-d_6/D_2SO_4) of **(2)** compared with samples of **(2)** exposed to I_2 vapours for a set numbers of days. Red circles denote resonances assigned to *trans,trans*-edb-I_4-H_2. The spectra were referenced by setting the aromatic proton doublet to 7.63 ppm, to counteract slight signal shifts due to differences in pH resulting from acid digestions

Although complicated by the presence of residual benzoic acid modulator, the ^{1}H NMR spectra (Figure S18) reveal that **(3)** is also reactive towards iodine vapours with new products obtained. A maximum value of 63% conversion of the alkyne bonds of bdb^{2-} to iodinated products was observed, although only minimally rising from the 59% that was obtained after 14 days. After 1 day, there is evidence of formation of partially iodinated bdb-I_2-H_2 alongside the fully iodinated *trans,trans*-bdb-I_4-H_2, with the majority of the iodination continuing to favour the formation of the fully iodinated ligand as the reaction time increases. The partially iodinated species is identifiable due to it lower symmetry, with some peaks coincident with the starting material and some with the fully iodinated linker. A maximum value of 88.5% *w/w* irreversible chemisorption of iodine was achieved.

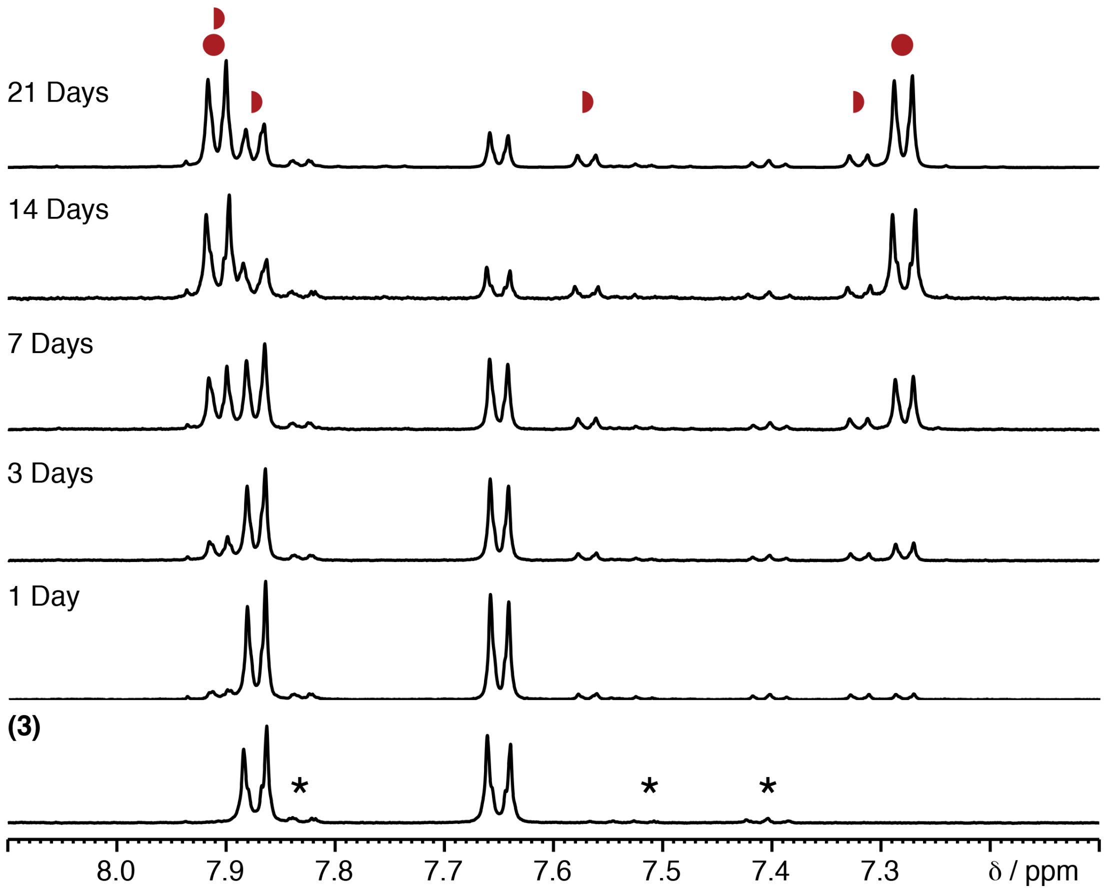

Figure S18. Stacked partial ^{1}H NMR spectra (DMSO-d_6/D_2SO_4) of **(3)** compared with samples of **(3)** exposed to I_2 vapours for a set numbers of days. Resonances marked with an asterix correspond to protons of residual benzoic acid from the synthesis of **(3)**, red semicircles denote resonances assigned to protons of the partially iodinated bdb-I_2-H_2 species, while red circles mark signals for the protons of *trans,trans*-bdb-I_4-H_2. The spectra were referenced by setting the aromatic proton doublet to 7.65 ppm, to counteract slight signal shifts due to differences in pH resulting from acid digestions.

The reactivity of **(4)** towards I_2 vapours (Figure S19) was initially slower than **(2)** or **(3)**, however the rate of iodination increased and appeared to remain constant over a longer period of time with no decrease in the rate of iodination observed until 21 days. Similar to the iodination of **(3)**, partially iodinated peb-I_2-H_2 alongside the fully iodinated *trans,trans*-peb-I_4-H_2 ligands was observed. Overall, 75% conversion of the alkyne units of peb^{2-} to iodinated species was detected, corresponding to 79% *w/w* irreversible chemisorption of iodine.

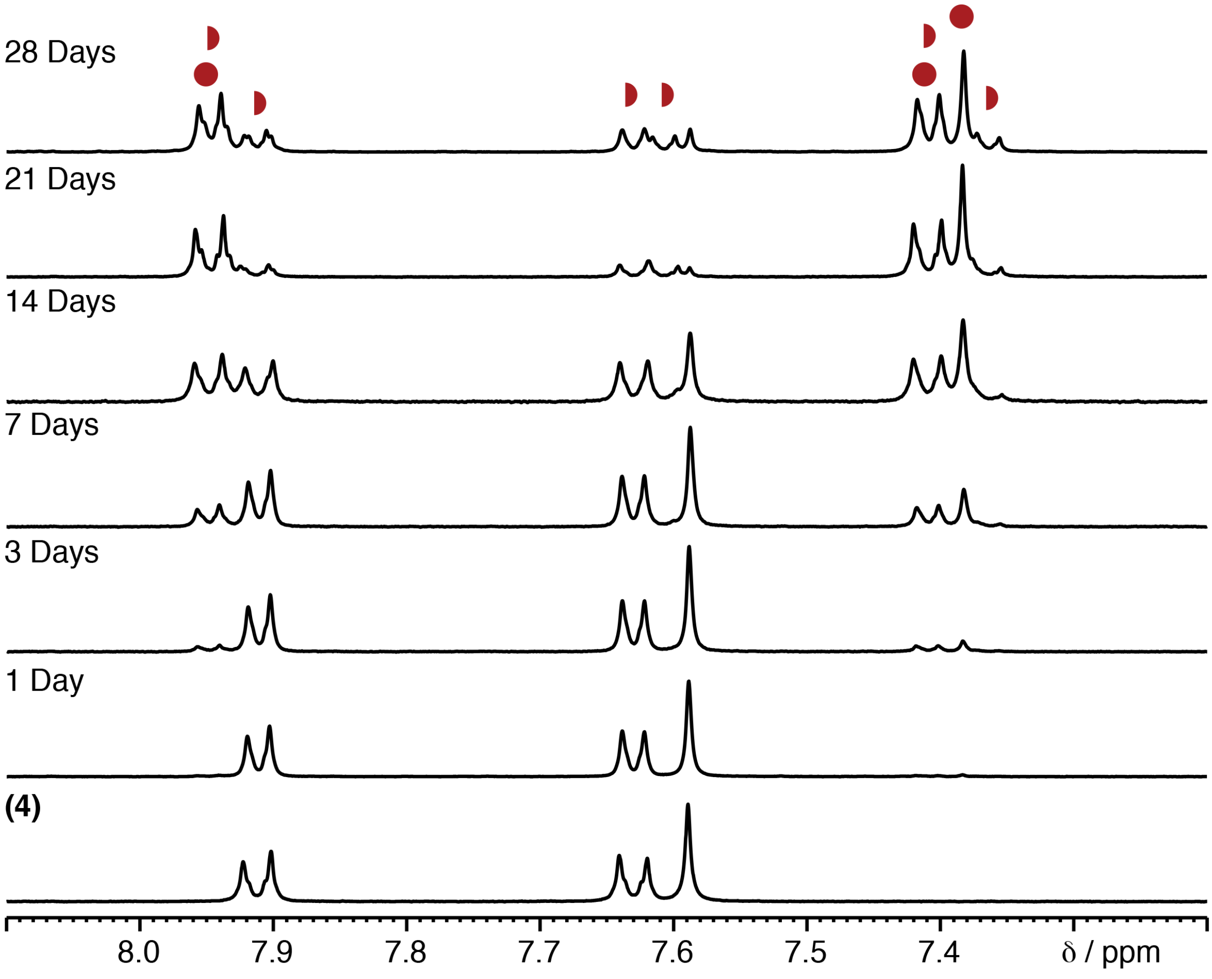

Figure S19. Stacked partial ^{1}H NMR spectra (DMSO-d_6/D_2SO_4) of **(4)** compared with samples of **(4)** exposed to I_2 vapours for a set numbers of days. Red semicircles denote resonances of protons of the partially iodinated peb-I_2-H_2, species while red circles mark signals of the protons of *trans,trans*-peb-I_4-H_2. The spectra were referenced by setting the aromatic proton doublet to 7.63 ppm, to counteract slight signal shifts due to differences in pH resulting from acid digestions.

A plot of iodination conversion versus time reveals clear differences in the reactivity of the four MOFs towards iodine vapours (Figure S20) which will influence their potential for use as iodine storage materials. The absence of iodinated products in the ^{1}H NMR spectra of digests of **(1)** is surprising, considering its high reactivity towards elemental bromine as well as NBS as a bromine source. The lack of reactivity of the alkene is in stark contrast to the alkyne units in **(2)**, **(3)** and **(4)**, which show total conversions in the 65-85% range after 21 days.

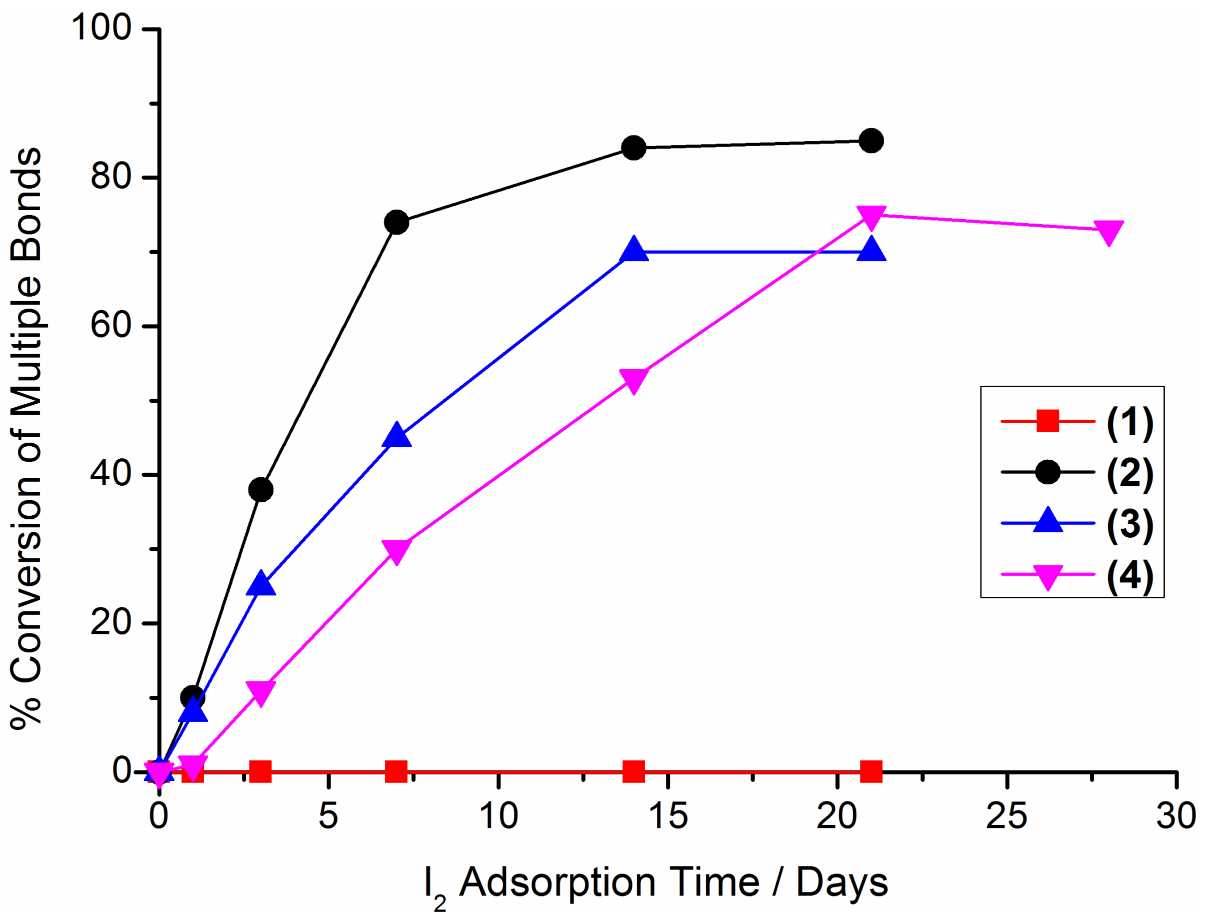

Figure S20. Comparison of the I_2 chemisorption capacity of each of the four MOFs investigated.

During centrifugation steps, it became apparent that there were large quantities of iodine being removed by solvent rinses, suggesting iodine physisorption within the MOF pores. Total iodine uptake measurements were then undertaken with data recorded over the same time range as the chemisorption experiments (although more data points were collected), allowing the total quantity of adsorbed iodine to be calculated.

Iodine Total Uptake Experiments

In a typical reaction, **(1)**, **(2)**, **(3)** or **(4)** (20 mg) was added to a pre-weighed 1 dram open-ended vial (Table S5). Separately, I_2 (15 eq) was added to a 30 ml screw top vial and the small vial containing the MOF was then placed in the centre of the large vial, with the lid of the outer vial tightened to create a closed system. The experiments were allowed to stand at room temperature with the small vial removed, weighed and placed back inside the large vial after 1, 3, 7, 14 and 21 days (data was also collected for the reaction containing **(4)** at 28 days). The gravimetric I_2 uptake of the MOFs was calculated from the observed increase in mass.

Table S5. Summary of reaction quantities for total I_2 uptake experiments.

**MOF**	**Mass (mg)**	**mmol unsaturated bond**	**mmol I_2**	**Mass I_2 (mg)**	**Equivalents I_2**
**(1)**	20	0.053	0.79	100	15
**(2)**	20	0.053	0.80	100	15
**(3)**	20	0.100	1.49	190	15
**(4)**	20	0.084	1.26	160	15

The simple experimental set-up was very efficient for determining the total iodine uptake capacity of the MOFs. Within a very short time (i.e. on the order of minutes) the MOF powders contained within the small vials were observed to darken, suggesting the diffusion of iodine occurred almost instantaneously. The uptake of iodine was monitored up to 21 days (28 days for the reaction containing **(4)**), and visualised by a plot of uptake versus time (Figure S21, overleaf).

A noticeable observation from the gravimetric I_2 uptake curves is the capability of **(1)** to physisorb I_2, yet no reaction occurs across the alkene bonds. There is a striking similarity between the weight % total I_2 uptake capacity of both **(2)** and **(3)**, which is not surprising considering the MOFs have very similar constituents, with **(3)** containing a slightly extended organic ligand. The I_2 uptake kinetics of the three non-interpenetrated MOFs are similar.

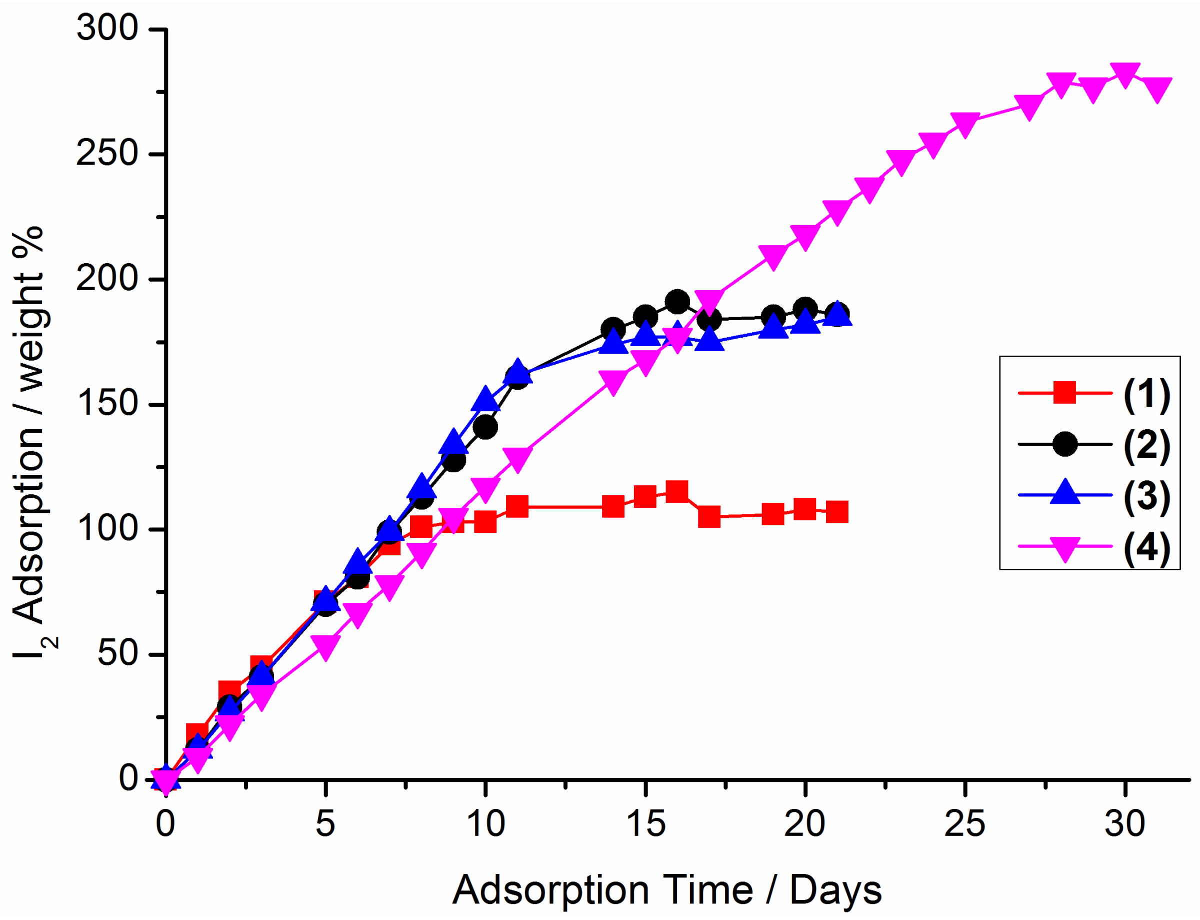

Figure S21. Comparison of the gravimetric iodine uptake capabilities of the MOFs when exposed to iodine vapour.

Again, similar to the chemisorption experiments it is clear that the uptake of I_2 by **(4)** is the slowest of the four MOFs, although it continues to adsorb I_2 for 28 days, which is the longest for the MOFs investigated. The ability of **(4)** to adsorb iodine for long periods of time results in an uptake performance that is by far the most superior, with a maximum uptake of 279% *w/w* recorded. The vast iodine uptake capacity of **(4)** is a result of both its ability to physisorb iodine within its pores, as well as irreversibly trapping it by chemical reaction across the alkyne units to convert them to diiodoalkene units. The chemisorptive capacity of **(4)** was actually lower than **(3)** when considered by weight %, therefore the superior uptake must be the result of a high tendency of I_2 to physisorb within the pores of **(4)**. A plausible reason for the increased I_2 physisorption may be the result of the interpenetrated nature of **(4)**, leading to an increased density of highly electron rich Zr_6 clusters. These results unambiguously highlight that zirconium MOFs are promising candidates as iodine capture/storage materials.

S11. Crystal Structures of Iodinated Linkers

The stereoselectivities of the iodination reactions were confirmed by the isolation of single crystals of iodinated linkers from digested samples (DMSO-d_6/D_2SO_4) of **(2)**, **(3)** and **(4)** that had been exposed to iodine vapours. In all cases, crystals of DMSO solvates separated from the solutions.

The crystal structure of *trans*-edb-I_2-H_2, which exhibits the expected *trans*-diiodoalkene geometry, was reported in our preliminary communication (CCDC deposition 1400977).[S8]

Data were collected using Collect[S19] and processed using SAINT v8.34A.[S20] The structure was solved using Superflip[S15] and refined with SHELXL[S12] within Olex2.[S13] All non-hydrogen atoms were refined with anisotropic adps with enhanced rigid-body (RIGU) and similarity restraints (SIMU) applied. Distance restraints were applied to C-C distances. Hydrogen atoms were placed at calculated positions and included in a riding model other than the methyl and OH hydrogen atoms which were refined as rigid-rotors. A region of poorly defined solvent was accounted for using SQUEEZE within PLATON[S14] which calculated a solvent accessible volume from two voids per unit cell of 327 Å^3 containing 138 electrons, potentially corresponding to approximately 6 molecules of DMSO per cell. The structure was deposited with the Cambridge Structural Database with deposition number CCDC 1443200.

Crystal Data for ***trans,trans*-bdb-I$_4$-H$_2$**. $C_{18}H_{10}I_4O_4{\cdot}C_2H_6OS$, M_r = 875.99, Monoclinic, a = 51.19 (7) Å, b = 7.307 (10) Å, c = 14.86 (2) Å, β = 100.67 (3)°, V = 5461 (13) Å^3, T = 100 K, space group $C2/c$ (no. 15), Z = 8, 13979 measured reflections, 4872 unique (R_{int} = 0.237), which were used in all calculations. The final R_1 = 0.123 for 1564 observed data $R[F^2 > 2\sigma(F^2)]$ and $wR(F^2)$ = 0.330 (all data).

Single crystal X-ray diffraction data were collected and processed using APEX2[S16] and the structure was solved using SHELXT and refined using SHELXL[S12] within Olex2.[S13] All non-hydrogen atoms were refined with anisotropic atomic displacement parameters (adps) with an enhanced rigid-body restraint (RIGU) applied. Hydrogen atoms were placed at calculated positions and included in a riding model other than the methyl and OH hydrogen

atoms which were refined as rigid-rotors. The structure was deposited with the Cambridge Structural Database with deposition number CCDC 1443201.

Crystal Data for ***trans,trans*-peb-I_4-H_2**. $C_{24}H_{14}I_4O_4 \cdot 2(C_2H_6OS)$, M_r = 1030.21, Triclinic, a = 5.8105 (9) Å, b = 6.7307 (10) Å, c = 22.342 (4) Å, α = 94.454 (5)°, β = 94.741 (5)°, γ = 108.638 (5)°, V = 820.1 (2) Å^3, T = 100 K, space group P-1 (no. 2), Z = 1, 10741 measured reflections, 2818 unique (R_{int} = 0.122), which were used in all calculations. The final R_1 = 0.105 for 2002 observed data $R[F^2 > 2\sigma(F^2)]$ and $wR(F^2)$ = 0.219 (all data).

S12. Dehalogenation of (1-Br_2)

Following our attempts to brominate **(1)** under a variety of conditions, we decided to try the reverse reaction and attempted dehalogenation of **(1-Br_2)** to regenerate **(1)**.[S21]

Dehalogenation of **(1-Br_2)**

(1-Br_2) (0.020 g, 0.037 mmol ligand, 1 eq) was added to a 14 ml vial and suspended in 2 ml acetone by stirring. Pyrollidine (46 µl, 0.56 mmol, 15 eq) was added and the vial was sealed and left to stir at room temperature for 42 hours. The reaction product **(1)** $[Zr_6O_4(OH)_4(sdc)_6]_n$ was collected by centrifugation and washed three times with acetone (10 ml), before being dried under vacuum. The extent of debromination was evident from the ^1H NMR spectrum of the digested (D_2SO_4/DMSO-d_6) reaction product (Figure S22).

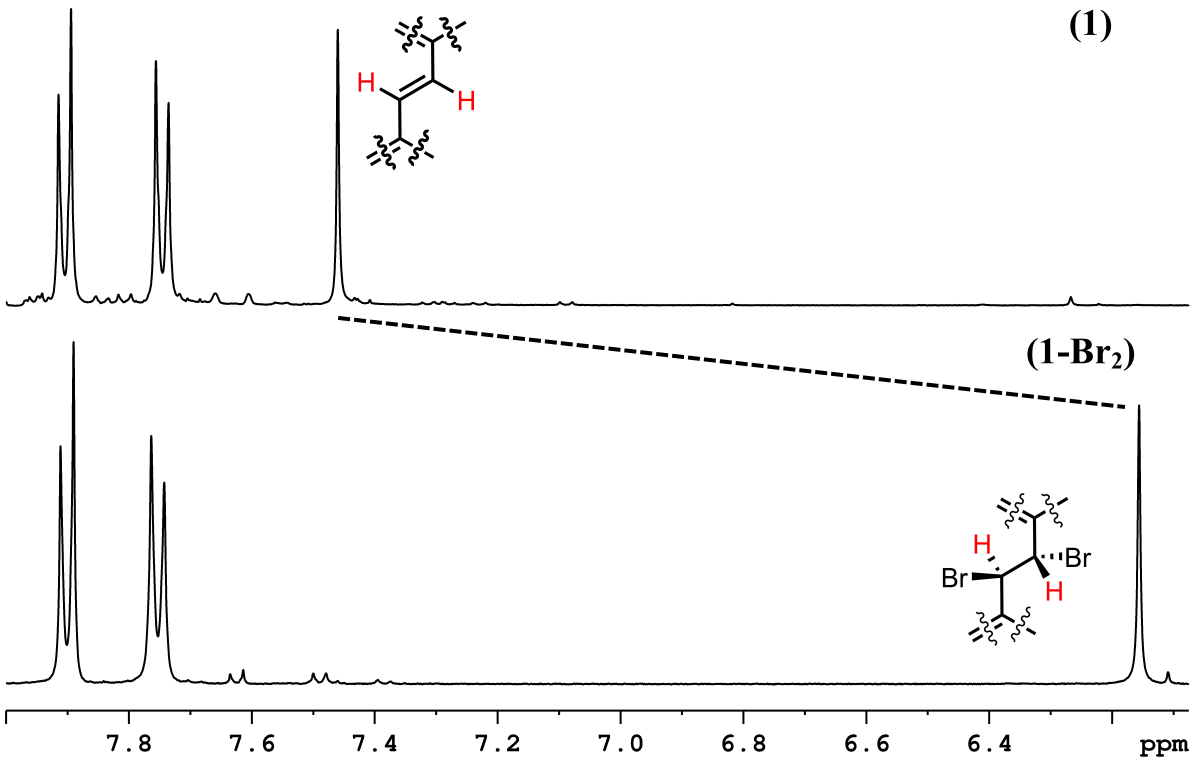

Figure S22. Partial ^1H NMR spectra (DMSO-d_6/D_2SO_4) of **(1-Br_2)** and **(1)** which is regenerated by debromination using pyrollidine. The spectra were referenced by setting the furthermost downfield aromatic doublet to 7.90 ppm, to counteract slight signal shifts due to differences in pH resulting from acid digestions.

Pyrollidine is able to successfully debrominate **(1-Br_2)**, resulting in quantitative conversion back to the parent MOF **(1)**. This is evident from the characteristic downfield shift of the alkene protons upon removal of the bromine atoms from the bromoalkane unit, regenerating an alkene. This transformation is an example of a tandem PSM event proving the high mechanical stability of these MOFs as the integral carbon atoms are observed to cycle from sp^2 to sp^3 and lastly back to sp^2.

S13. References

[S1] C. F. Macrae, I. J. Bruno, J. A. Chisholm, P. R. Edgington, P. McCabe, E. Pidcock, L. Rodriguez-Monge, R. Taylor, J. van de Streek and P. A. Wood, *J. Appl. Cryst.*, **2008**, *41*, 466–470.

[S2] T. Gadzikwa, B.-S. Zeng, J. T. Hupp and S. T. Nguyen, *Chem. Commun.*, **2008**, 3672–3674.

[S3] M. G. Vivas, D. L. Silva, L. De Boni, Y. Bretonniere, C. Andraud, F. Laibe-Darbour, J. C. Mulatier, R. Zaleśny, W. Bartkowiak, S. Canuto and C. R. Mendonca, *J. Phys. Chem. B,* **2012**, *116*, 14677–14688.

[S4] G. Zhang, H. Yi, G. Zhang, Y. Deng, R. Bai, H. Zhang, J. T. Miller, A. J. Kropf, E. E. Bunel and A. Lei, *J. Am. Chem. Soc.*, **2014**, *136*, 924–926.

[S5] L. M. Ballesteros , S. Martín , J. Cortés , S. Marqués-González, S. J. Higgins, R. J. Nichols, P. J. Low and P. Cea, *Chem. Eur. J.*, **2013**, *19*, 5352–5363.

[S6] D. Frahm, F. Hoffmann and M. Fröba, *Cryst. Growth Des.*, **2014**, *14*, 1719–1725.

[S7] R. J. Marshall, T. Richards, C. Hobday, C. F. Murphie, C. Wilson, S. A. Moggach, T. D. Bennett and R. S. Forgan, *Dalton Trans.*, **2016**, DOI: 10.1039/C5DT03178H.

[S8] R. J. Marshall, S. L. Griffin, C. Wilson and R. S. Forgan, *J. Am. Chem. Soc.*, **2015**, *137*, 9527–9530.

[S9] A. Schaate, P. Roy, T. Preuße, S. J. Lohmeier, A. Godt and P. Behrens, *Chem. Eur. J.*, **2011**, *17*, 9320–9325.

[S10] Rigaku (**2012**). CrystalClear-SM Expert 3.1 b27.

[S11] Agilent (**2014**). *CrysAlis PRO* Version 1.171.37.35 (release 13-08-2014 CrysAlis171.NET) Agilent Technologies Ltd, Yarnton, Oxfordshire, England.

[S12] G. M. Sheldrick, *Acta Cryst. C*, **2015**, *71*, 3–8.

[S13] O. V. Dolomanov, L. J. Bourhis, R. J. Gildea, J. A. K. Howard and H. Puschmann, *J. Appl. Cryst.* **2009**, *42*, 339–341

[S14] A. L. Spek, *Acta Cryst. C*, **2015**, *71*, 9–18.

[S15] L. Palatinus and G. Chapuis, *J. Appl. Cryst.* **2007**, *40*, 786–790.

[S16] Bruker (**2007**). Bruker AXS Inc., Madison, Wisconsin, USA.

[S17] S. Bar, *Can. J. Chem.*, **2010**, *88*, 605–612.

[S18] T. D. Bennett, P. J. Saines, D. A. Keen, J. –C. Tan and A. K. Cheetham, *Chem. Eur. J.* **2013**, *19*, 7049–7055.

[S19] Bruker (**2007-2014**). Bruker AXS Inc., Madison, Wisconsin, USA.

[S20] Bruker (**2013**). Bruker AXS Inc., Madison, Wisconsin, USA.

[S21] L. Luo, D. Resch, C. Wilhelm, C. N. Young, G. P. Halada, R. J. Gambino, C. P. Grey, and N. S. Goroff, *J. Am. Chem. Soc.*, **2011**, *133*, 19274–19277.
